# STING activation disrupts tumor vasculature to overcome the EPR limitation and increase drug deposition

**DOI:** 10.1126/sciadv.ado0082

**Published:** 2024-07-17

**Authors:** Xiaomin Jiang, Taokun Luo, Kaiting Yang, Morten J. Lee, Jing Liu, Langston Tillman, Wenyao Zhen, Ralph R. Weichselbaum, Wenbin Lin

**Affiliations:** ^1^ Department of Chemistry, The University of Chicago, 929 E 57th St, Chicago, IL 60637, USA.; ^2^ Department of Radiation and Cellular Oncology and Ludwig Center for Metastasis Research, The University of Chicago, 5758 S Maryland Ave., Chicago, IL 60637, USA.

## Abstract

The low success rate of cancer nanomedicines has raised debate on the role of the enhanced permeability and retention (EPR) effect on tumor deposition of nanotherapeutics. Here, we report a bifunctional nanoscale coordination polymer (NCP), oxaliplatin (OX)/2′,3′-cyclic guanosine monophosphate–adenosine monophosphate (GA), to overcome the EPR limitation through stimulator of interferon genes (STING) activation and enhance chemotherapeutic and STING agonist delivery for tumor eradication. OX/GA encapsulates GA and OX in the NCP to protect GA from enzymatic degradation and improve GA and OX pharmacokinetics. STING activation by OX/GA disrupts tumor vasculatures and increases intratumoral deposition of OX by 4.9-fold over monotherapy OX-NCP. OX/GA demonstrates exceptional antitumor effects with >95% tumor growth inhibition and high cure rates in subcutaneous, orthotopic, spontaneous, and metastatic tumor models. OX/GA induces immunogenic cell death of tumor cells and STING activation of innate immune cells to enhance antigen presentation. NCPs provide an excellent nanoplatform to overcome the EPR limitation for effective cancer therapy.

## Teaser

Nanoparticle-induced STING activation disrupts tumor vasculature to overcome the EPR limitation and increase drug deposition.

## INTRODUCTION

Immunotherapy has revolutionized cancer treatment (*1*). Monoclonal antibodies targeting the immune checkpoints have found clinical success in patients with immunogenic cancers such as melanoma and non–small cell lung cancer (*2*–*5*). However, activation of the adaptive immune system via immune checkpoint blockade (ICB) has not been ineffective against nonimmunogenic tumors with immunosuppressive tumor microenvironment (TME) and inadequate T cell infiltration (*6*, *7*). Chemotherapy regimens have been combined with anti-programmed cell death-1 (anti–PD-1) antibodies to enhance the antitumor efficacy of ICB in multiple tumor types with low programmed cell death ligand1 (PD-L1) expression (*8*, *9*).

Chemotherapy is an important modality for cancer treatment, but its efficacy is limited by the intratumoral drug concentration at tolerable doses (*8*, *10*). Because of their suboptimal pharmacokinetics (PK) and high accumulation in vital organs, chemotherapeutics often cause severe adverse effects such as neutropenia (*11*), hepatotoxicity (*12*), and renal toxicity (*13*, *14*). Nanomedicines aim to overcome these limitations by encapsulating chemotherapeutics into nanoparticles to enhance PK, prevent degradation, and prolong circulation of the drug cargoes (*15*, *16*). The enhanced permeability and retention (EPR) effect was postulated to explain nanoparticles’ ability to increase drug deposition in tumor tissues over free chemotherapeutics in preclinical models (*17*, *18*). The EPR effect results from passive accumulation of long-circulating nanoparticles in tumors owing to the characteristic leaky vasculature and ineffective lymphatic drainage in the TME (*19*). Nanoparticles in the size range of 10 to 200 nm can avoid clearance via renal filtration (<10 nm) and by the mononuclear phagocytic system (MPS) (>200 nm) and accumulate in tumors via the EPR effect (*20*, *21*). However, the low success rate of cancer nanomedicines has raised debate on the role of the EPR effect in enhancing drug deposition in tumors and the prevalence of the EPR effect in human tumors. The tumor vasculature is highly heterogeneous with variable vascular permeability, vascular maturation at the vessel level (*22*), extracellular matrix (*23*), and interstitial fluid pressure (*24*). PK improvement by nanoparticles does not sufficiently alleviate systemic deposition of chemotherapeutics and general toxicity. Thus, there is an unmet need for clinically relevant strategies that can modulate and remodel vastly heterogeneous TMEs, particularly tumor vasculatures, to increase tumor deposition and improve therapeutic indices of chemotherapeutics.

The stimulator of interferon genes (STING) axis mediates inflammation upon cellular stress or infection and senses pathogenic nucleotides to activate the innate immune response (*25*, *26*). Double-stranded DNA allosterically modulates cyclic guanosine monophosphate–adenosine monophosphate (GMP-AMP) synthase catalytic activity to produce 2′,3′-cyclic GMP-AMP (GA) in the cytoplasm, which can then bind STING dimers in the endoplasmic reticulum to cause STING oligomerization and trafficking, inducing the secretion of type I interferons (IFNs) and other proinflammatory cytokines (*27*). Hyperactive STING activation was shown to cause an autoimmune disease characterized by severe vasculitis and vasculopathy (*28*). STING is expressed in malignant and healthy cells in the TME, including endothelial cells (ECs), platelets, and the high-endothelial venule, which together form tumor vasculature. STING activation can profoundly disrupt abnormal vasculature patterns in the TME (*29*). Therefore, we hypothesized that STING agonists could provide a strategy to overcome the limitations of the EPR effect to markedly increase tumor deposition of chemotherapeutics by proactively disrupting the tumor vasculature. However, potent cyclic dinucleotide (CDN) STING agonists such as GA are quickly cleared from blood circulation due to rapid enzymatic degradation and poor PK profiles (*30*–*32*). Intratumoral injection of CDN STING agonists such as ADU-S100 and MK-1454 has demonstrated modest clinical responses, owing to their poor retention in tumors (*33*, *34*).

Here, we report the design of a nanoscale coordination polymer (NCP) encapsulating both GA and chemotherapeutic oxaliplatin (OX), OX/GA, to overcome the EPR limitation and markedly increase drug deposition in tumors through STING-mediated disruption of tumor vasculature. By protecting GA in the coordination polymer core, OX/GA prolonged blood circulation of GA to potently activate STING in tumor ECs for tumor vasculature disruption, thereby enhancing tumor accumulation of GA and OX. High concentrations of intratumoral GA and OX induced multiple immunostimulatory pathways and potent immunogenic cell death (ICD), respectively. Systemically administered OX/GA increased the tumor area under the curve (AUC) of OX by 4.9-fold over OX monotherapy nanoparticle (OX-NCP) and exhibited strong antitumor effect in subcutaneous, orthotopic, spontaneous, and metastatic tumor models with large tumor growth inhibition (TGI) values (>95%) and high cure rates. OX/GA stimulated the innate immune response, repolarized tumor-associated macrophages (TAMs), enhanced tumor-associated antigen (TAA) presentation, and sensitized immunologically “cold” tumors to ICB.

## RESULTS

### Formulation of OX/GA

**Fig. 1.**
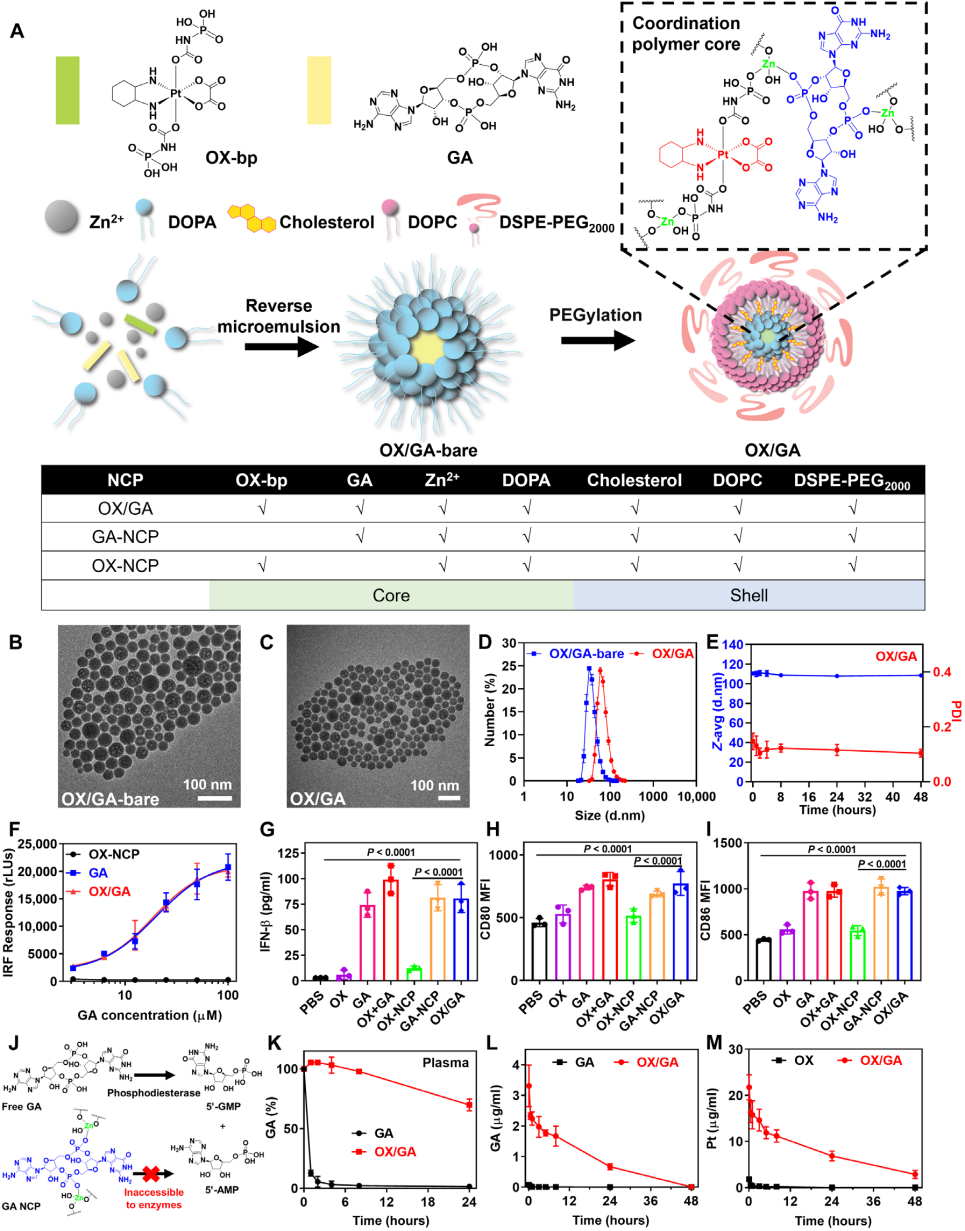
OX/GA activates STING and prolongs blood circulation of GA. (**A**) Schematic illustration of OX/GA with a coordination polymer core of GA, OX-bp, and Zn^2+^ and a lipid shell of DOPA, cholesterol, DOPC, and DSPE-PEG_2000_. The table illustrates the compositions of NCP treatments. (**B** and **C**) TEM images of OX/GA-bare and OX/GA. (**D** and **E**) Size distributions of OX/GA-bare and OX/GA (D) and OX/GA in BSA (5 mg/ml) at 37°C (E) by DLS (*n* = 3). d.nm., diameter values in nanometers. (**F**) Dose-dependent IRF responses of THP-1 reporter cells (*n* = 3). RLU, relative light units. (**G**) Type I IFN secretion from BMDCs after 24-hour incubation with drugs at a dose of 15 μM OX and/or 1 μM GA (*n* = 3). (**H** and **I**) Maturation of BMDCs treated with OX/GA for 24 hours by CD80 up-regulation and CD86 up-regulation (*n* = 3). (**J**) Schematic showing how OX/GA protects GA from enzymatic degradation. (**K**) Stability of GA for GA or OX/GA incubated in rat plasma at 37°C (*n* = 3). (**L** and **M**) GA or Pt PK profiles after intravenous injection of OX, GA, or OX/GA to rats (*n* = 3). Data in (D) to (I) and (K) to (M) are presented as means ± SD. *P* values in (G) to (I) are analyzed by one-way ANOVA with Tukey’s multiple comparisons tests.

We synthesized OX/GA in two steps. First, the phosphate groups of the OX prodrug Pt(dach)(oxalate)(bisphosphoramidic acid) (OX-bp) (*35*) and GA were cross-linked with Zn^2+^ cations in a reverse microemulsion with Triton X-100/hexanol/cyclohexane in the presence of 1,2-dioleoyl-*sn*-glycero-3-phosphate (DOPA). The polar lipid DOPA capped the particles to form OX/GA-bare particles with a hydrophobic surface (Fig. 1A). Next, OX/GA-bare was coated with a mixture of 1,2-dioleyl-*sn*-glycero-3-phosphocholine (DOPC), cholesterol, and 1,2-distearoyl-*sn*-glycero-3-phosphoethanolamine-*N*-[amino(polyethylene glycol)2000] (DSPE-PEG_2000_) via hydrophobic/hydrophobic interactions to form OX/GA NCP. OX/GA with an OX:GA molar ratio of 15:1 was used for all studies in this work based on an in vivo screening study, which is discussed below. The loadings of OX and GA in OX/GA were determined to be 8.4 ± 0.7 and 1.1 ± 0.1%, respectively.

OX/GA-bare and OX/GA particles showed spherical morphology (Fig. 1, B and C), with *Z*-average diameters of 61.2 ± 0.2 and 110.7 ± 1.7 nm, respectively, and polydispersity indices (PDIs) of 0.12 ± 0.01 and 0.15 ± 0.03, respectively (Fig. 1D and fig. S1). OX/GA-bare showed high stability in tetrahydrofuran (THF) at room temperature, and OX/GA particles were stable in phosphate-buffered saline (PBS) containing bovine serum albumin (BSA) (5 mg/ml) with no changes in sizes and PDIs at 37°C over 48 hours (Fig. 1E and fig. S1). The monotherapy control particles with only OX-bp (OX-NCP) and only GA (GA-NCP) in the core were prepared by omitting GA and OX-bp, respectively, in their synthesis. The control particles used in the present study were similarly prepared and characterized (fig. S1).

### OX/GA elicits STING activation

OX/GA was found to induce STING activation. THP-1 monocyte cells with an incorporated IFN-stimulated gene for STING reporting were treated with OX-NCP, GA, or OX/GA (Fig. 1F). While OX-NCP had no obvious IFN regulatory factor (IRF) response, OX/GA and GA showed similar median effective concentration (EC_50_) values of 20.7 ± 1.2 and 23.8 ± 1.3 μM, respectively. Bone marrow-derived dendritic cells (BMDCs) from C57BL/6 mice were next used as an ex vivo indicator for STING activation. After incubation with BMDCs for 24 hours, OX/GA induced a similar level of IFN-β secretion to either GA or OX plus GA (OX+GA; Fig. 1G). STING activation by OX/GA led to maturation of BMDCs (Fig. 1, H and I). After 24-hour incubation, OX/GA-treated DCs up-regulated CD80 by 1.7-fold and CD86 by 2.2-fold over PBS control. Treatment with OX+GA led to similar CD80 and CD86 up-regulation as OX/GA. These results indicate that OX/GA efficiently activates STING signaling.

### OX/GA prevents GA degradation and improves PK

Clinical translation of CDN STING agonists such as GA is hindered by rapid pyrophosphatase-mediated and phosphodiesterase-mediated degradation, which prevents systemic delivery of CDNs (Fig. 1J) (*36*). We compared GA stability of OX/GA or free GA in rat plasmas over time. OX/GA markedly slowed GA degradation and prolonged the GA half-life to >24 hours from 0.14 hours for free GA (Fig. 1K). This result shows that OX/GA effectively protects GA from enzymatic degradation.

OX/GA significantly enhanced the PK of GA and OX in Sprague-Dawley (SD) rats. While free GA and OX only showed a plasma half-life of 0.29 and 0.21 hours, respectively, OX/GA extended serum half-lives of GA and OX to 16.33 and 19.94 hours, respectively (Fig. 1, L and M, and tables S1 and S2). OX/GA significantly increased GA and Pt blood exposure with AUC_0-inf_ values of 42.72 ± 4.03 and 454.76 ± 71.46 (μg/ml)·hour, respectively, which were 250-fold larger than that of GA [with an AUC_0-inf_ of 0.17 ± 0.01 (μg/ml)·hour] and 132-fold larger than that of OX [with an AUC_0-inf_ of 3.44 ± 0.19 (μg/ml)·hour]. These results demonstrate that NCP provides an effective strategy to prolong GA and OX circulation in the blood by preventing enzymatic degradation and avoiding clearance by the MPS.

### OX/GA disrupts tumor vasculature to enhance drug deposition

**Fig. 2.**
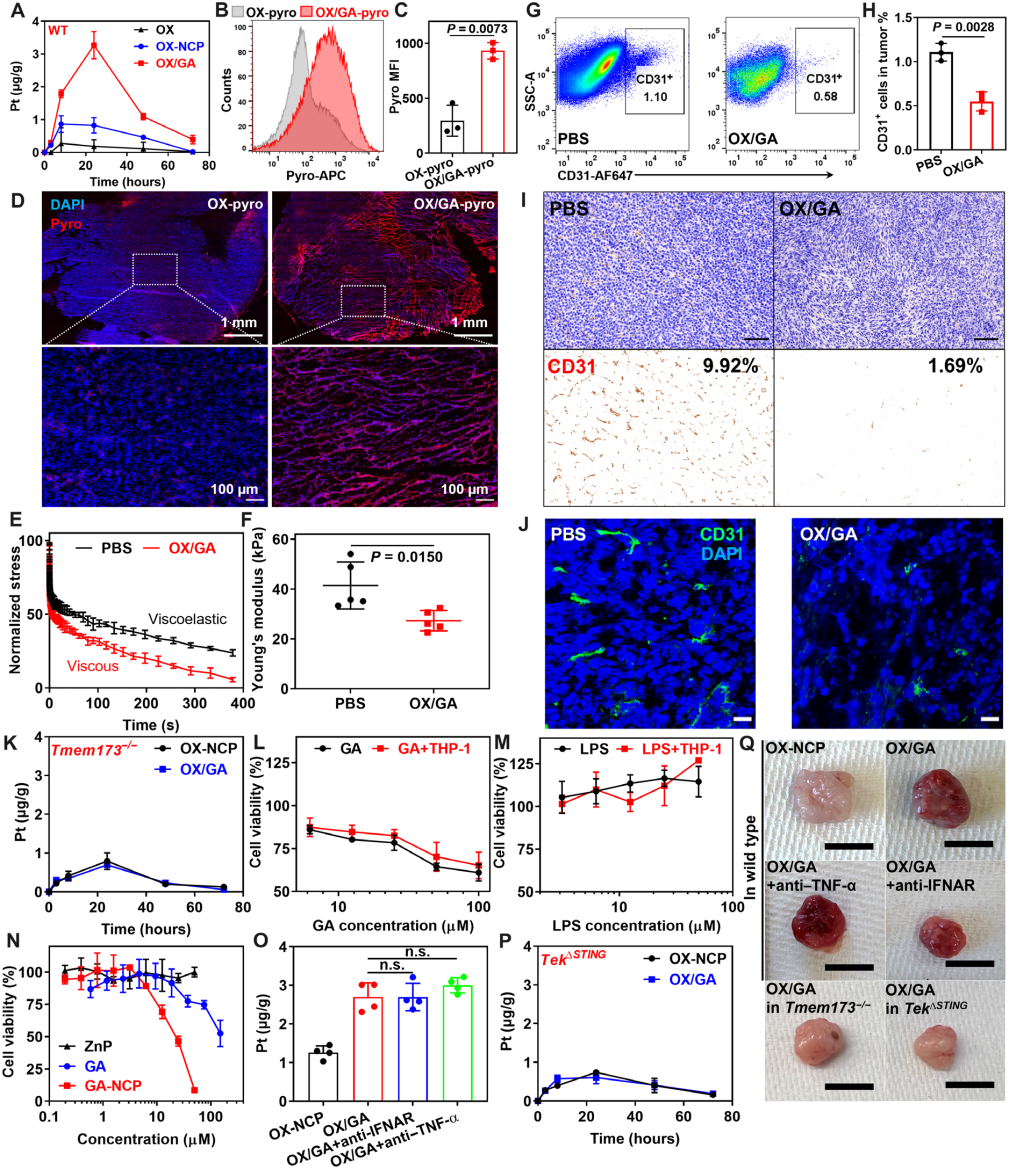
STING activation disrupts tumor vasculature and enhances drug deposition in tumors. (**A**) Pt levels in MC38 tumors in C57BL/6 mice after intravenous injection of OX, OX-NCP, and OX/GA (*n* = 3). (**B** and **C**) Flow cytometry and quantitative fluorescence analysis for OX-pyro and OX/GA-pyro 24 hours after intravenous injection (*n* = 3). (**D**) Immunofluorescence staining for OX-pyro and OX/GA-pyro in MC38 tumors 24 hours after intravenous injection. (**E** and **F**) Normalized stress (E) and Young’s modulus (F) of MC38 tumors after OX/GA treatment [(E), *n* = 3; (F), *n* = 5]. (**G** and **H**) Flow cytometry and quantitative fluorescence analysis of CD31^+^ ECs of MC38 tumors after OX/GA treatment (*n* = 3). SSC-A, side scatter area. (**I** and **J**) CD31 IHC staining and CD31 immunofluorescence imaging of MC38 tumors after OX/GA treatment. Scale bars, 200 μm in (I) and 40 μm in (J). (**K**) Pt levels in MC38 tumors from *Tmem173^−/−^* mice treated with OX-NCP and OX/GA (*n* = 3). (**L** and **M**) MTS assays of HUVECs treated with GA or LPS with or without coincubation of THP-1 cells. (**N**) MTS assays of GA-NCP, GA, and ZnP in HUVECs (*n* = 3). (**O**) Tumor accumulation of Pt 24 hours after intravenous injection of OX-NCP or OX/GA and intraperitoneal injection of 500 μg of anti-IFNAR or anti–TNF-α antibody (*n* = 4). (**P**) Pt levels in MC38 tumors in *Tek*^ΔSTING^ mice treated with OX-NCP or OX/GA (*n* = 3). (**Q**) Representative photos of subcutaneous MC38 tumors in C57BL/6, *Tmem173^−/−^*, or *Tek*^ΔSTING^ mice 24 hours after treatment. Scale bars, 1 cm. Data in (A), (C), (E), (F), (H), and (K) to (P) are presented as means ± SD. *P* values in (O) are analyzed by one-way ANOVA with Tukey’s multiple comparisons tests. Unpaired *t* tests were used to compare the two groups in (C), (F), and (H).

To examine the biodistribution and tumor accumulation of OX/GA, we analyzed Pt contents in tumors by inductively coupled plasma mass spectrometry (ICP-MS) after intravenous injection of OX-NCP or OX/GA at 3.0 mg OX/kg and 0.36 mg GA/kg to MC38 tumor-bearing C57BL/6 mice. In the tumors, OX/GA showed a fourfold higher Pt concentration than OX-NCP at 24 hours after injection and increased Pt AUC by 4.9-fold over OX-NCP [290.3 versus 59.7 (μg/ml)·hour; Fig. 2A] and 20.7-fold over OX [14.0 (μg/ml)·hour]. Meanwhile, OX-NCP and OX/GA showed similar plasma PK and similar levels of accumulation in the major organs such as the hearts, lungs, livers, spleens, and kidneys (fig. S2), indicating that OX/GA specifically enhances drug deposition in the tumors.

To track nanoparticle uptake and localization, we coated OX-NCP and OX/GA with cholesterol-pyropheophytin (Chol-pyro) to afford fluorescently labeled OX-pyro and OX/GA-pyro particles, respectively (ex/em, 665 nm/674 nm; fig. S3). We determined pyro uptake by tumor cells 24 hours after intravenous injection of OX-pyro and OX/GA-pyro by flow cytometry (Fig. 2, B and C). OX/GA-pyro showed 3.2-fold higher pyro signal than OX-pyro, which supported the enhanced tumor uptake of OX from OX/GA by ICP-MS analysis. Immunofluorescence staining also showed that OX/GA-pyro treatment exhibited a much higher pyro signal in the tumor (Fig. 2D). OX/GA-pyro treatment also changed the tumors from a dense cellular arrangement to a loose and leaky pattern.

To confirm this observation, we conducted stress relaxation measurements on excised MC38 tumors. Tumors treated with PBS displayed viscoelastic properties, indicating a mechanical response characterized by both elastic and viscous features during deformation. In contrast, OX/GA-treated tumors exhibited a substantial reduction in elasticity while displaying increased viscosity, suggesting a shift in tumor biomechanics (Fig. 2E). In addition, we assessed Young’s modulus, a mechanical parameter that reflects the stiffness of solid materials, through uniaxial compression of MC38 tumors (Fig. 2F). At 24 hours after treatment, OX/GA caused a notable decrease in Young’s moduli of MC38 tumors, reducing them to 27.3 kPa from 41.4 kPa for untreated tumors. These findings indicate that OX/GA effectively reduces tumor stiffness and modulates tumor biomechanics. This morphology change may increase tumor permeability, which can not only enhance drug deposition but also facilitate immune cell infiltration into tumors (*37*).

We hypothesized that OX/GA-induced enhanced drug deposition in tumors was related to the EPR effect. The EPR theory attributes intratumoral accumulation of nanoparticles to the leaky tumor vasculature. We assessed the tumor vessel density through CD31 staining 24 hours after intravenous injection of OX/GA in MC38 tumor-bearing C57BL/6 mice. Flow cytometry analysis revealed a twofold reduction of CD31^+^ ECs in the tumors (Fig. 2, G and H). Immunohistochemistry (IHC) staining of CD31 further demonstrated that GA-NCP and OX/GA treatments significantly decreased vessel density to 1.87 and 1.69%, respectively, from 9.92% in the PBS group (Fig. 2I and fig. S4). Notably, treatment with OX+GA or OX-NCP did not result in obvious changes in vessel density (fig. S4). CD31 immunofluorescence staining also confirmed a notable reduction in tumor vasculature in GA-NCP and OX/GA-treated tumors (Fig. 2J and fig. S4). We also assessed vessel density in the liver and spleen and found that OX/GA did not induce obvious vasculature disruption in either the liver or the spleen (fig. S5). These findings indicate that GA-loaded NCPs disrupt tumor vasculature to reduce vessel density and increase vasculature permeability for enhanced drug deposition in tumors.

### OX/GA induces tumor vasculature disruption via endothelial STING activation

We next determined the mechanism of OX/GA-induced tumor vasculature disruption. We first evaluated the tumor deposition of OX/GA in MC38 tumor-bearing *Tmem173^−/−^* (STING knockout) mice. In contrast to wild-type (WT) B6 mice (Fig. 2A), OX/GA did not increase Pt deposition in tumors over OX-NCP (Fig. 2K), indicating the OX/GA-induced tumor vasculature disruption depends on STING activation of the host.

OX/GA-induced tumor vasculature disruption can occur through EC STING activation or acute elevation of cytokines secreted by other STING-activated cell populations (fig. S6) (*38*). To distinguish between these two possibilities, we cocultured human umbilical vein ECs (HUVECs) and THP-1 cells in the presence of different concentrations of GA or the positive control lipopolysaccharide (LPS). 3-(4,5-Dimethylthiazol-2-yl)-5-(3-carboxymethoxyphenyl)-2-(4-sulfophenyl)-2*H*-tetrazolium (MTS) assays showed that the viability of HUVECs decreased in a GA concentration-dependent manner, but the addition of THP-1 monocytes did not enhance the cytotoxicity (Fig. 2L). LPS treatment did not cause toxicity to HUVECs with or without coincubation of THP-1 cells (Fig. 2M), suggesting that proinflammatory cytokines secreted by THP-1 cells did not cause cytotoxicity to ECs. The cytotoxicity of GA in HUVECs was further amplified 7.7 times through formulation into GA-NCP (Fig. 2N and fig. S6). This enhanced potency was attributed to the improved GA delivery by GA-NCP due to the stabilization of GA against enzymatic degradation in physiological environments (Fig. 1, J and K). Moreover, GA-NCP treatment increased apoptosis and necrosis, as evidenced by the stronger signal observed in annexin V and propidium iodide staining by flow cytometry (fig. S6). These results suggest that direct activation of endothelial STING, rather than cytokine elevation, is the major contributor to STING-mediated endothelial cytotoxicity.

To validate our hypothesis in vivo, we determined tumor deposition of OX/GA in MC38 tumor-bearing C57BL/6 mice with cytokine blocking antibodies and in MC38 tumor-bearing *Tek*^ΔSTING^ mice (EC-specific STING conditional knockout mice). Intraperitoneal injection of an anti-interferon alpha receptor (anti-IFNAR) antibody or an anti–tumor necrosis factor–α (TNF-α) antibody did not reduce Pt deposition in OX/GA-treated tumors (Fig. 2O). In contrast, OX/GA exhibited similar intratumoral Pt concentration as OX-NCP in MC38-bearing *Tek*^ΔSTING^ mice, which was significantly lower than in WT mice (Fig. 2P). In the excised tumors, OX/GA caused the appearance of bloody tumors 24 hours after treatment in WT mice, but OX-NCP treatment did not show the same phenomenon (Fig. 2Q). Type I IFN or TNF-α blockade did not affect the bloody appearance of OX/GA-treated tumors. However, the appearance of bloody tumors was alleviated in *Tmem173^−/−^* mice and *Tek*^ΔSTING^ mice (Fig. 2Q). These results strongly support that OX/GA-induced tumor vasculature disruption is dependent on endothelial STING activation.

### Systemic delivery of OX/GA eradicates tumors

We first evaluated the anticancer efficacy of OX/GA on subcutaneous MC38 and CT26 murine colorectal tumor models in C57BL/6 and BALB/c mice, respectively. We first determined the optimal ratio between OX and GA and their respective doses (3.0 mg/kg OX and 0.36 mg/kg GA) based on an in vivo screening of mouse body weight changes after continuous treatment with different doses of monotherapy particles GA-NCP and OX-NCP once every 3 days (Q3D; fig. S7). Mice were intravenously injected with PBS, OX+GA, OX-NCP, GA-NCP, or OX/GA at equivalent OX of 3.0 mg/kg and/or 0.36 mg GA/kg Q3D.

**Fig. 3.**
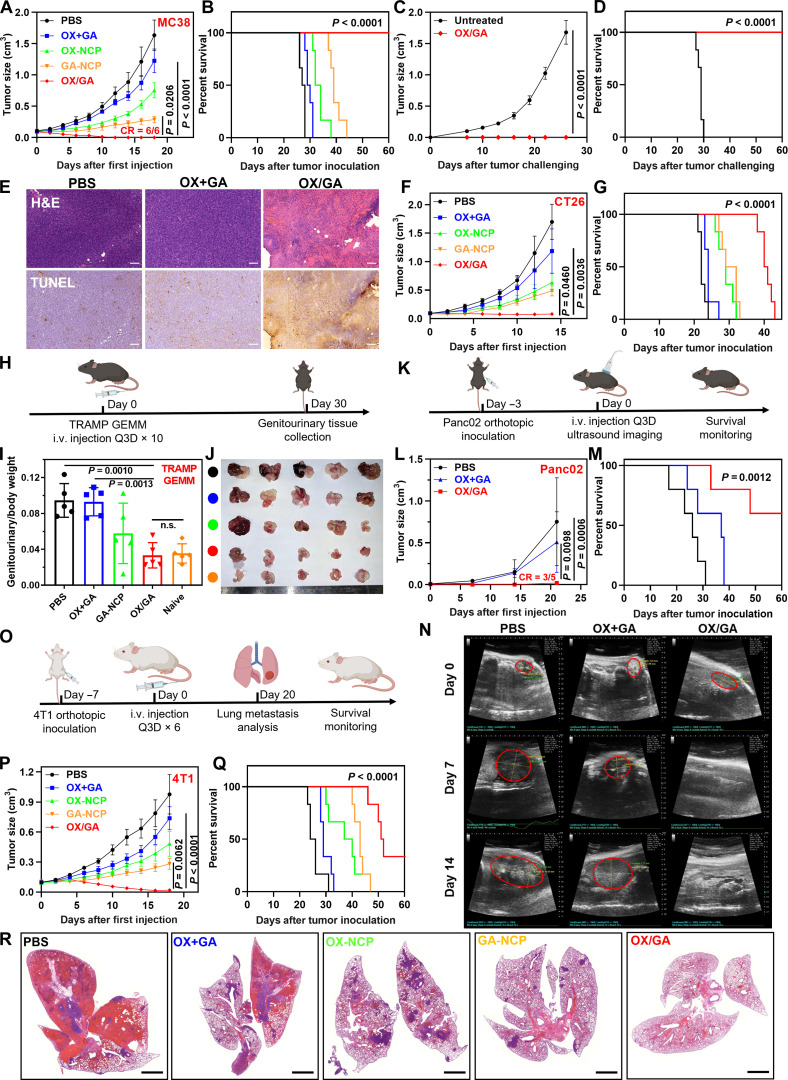
In vivo antitumor efficacy. (**A** and **B**) Tumor growth curves and survival curves of MC38 tumor-bearing C57BL/6 mice after intravenously injected with PBS, OX+GA, OX-NCP, GA-NCP, or OX/GA at 3.0 mg OX /kg and/or 0.36 mg GA/kg Q3D for five doses in total (*n* = 6). (**C** and **D**) Tumor growth curves and survival curves of C57BL/6 mice receiving MC38 challenge after indicated treatments (*n* = 6). (**E**) H&E and TUNEL staining of excised tumors after indicated treatments. (**F** and **G**) Tumor growth curves and survival curves of CT26 tumor-bearing BALB/c mice after indicated treatments for five doses (*n* = 6). (**H**) Dosing schedule for TRAMP mice and genitourinary tissue collection. i.v., intravenous. (**I** and **J**) Ratios of genitourinary tract weights over total body weights (I) and photos (J) of naive mice and TRAMP mice after indicated treatments for 10 doses (*n* = 5). (**K**) Dosing and imaging schedule for orthotopic Panc02 tumor-bearing C57BL/6 mice. (**L** and **M**) Tumor growth curves and survival curves of orthotopic Panc02 tumor-bearing C57BL/6 mice after indicated treatments for four doses (*n* = 5). (**N**) Tumor ultrasound imaging was performed weekly on days 0, 7, 14, and 21 after the first injection. (**O**) Dosing schedule for 4T1 orthotopic model and lung metastasis analysis. (**P** and **Q**) Tumor growth curves and survival curves of orthotopic 4T1 tumor-bearing BALB/c mice after indicated treatments for six doses (*n* = 6). (**R**) H&E staining for lung metastasis after indicated treatments. Scale bars, 2 mm. Data in (A), (C), (F), (I), (L), and (P) are presented as means ± SD. The data were analyzed by one-way ANOVA with Tukey’s multiple comparisons tests. A log-rank (Mantel-Cox) test was used for the statistical analysis of survival curves. The schemes in (H), (K), and (O) were created with BioRender.com.

Following five injections of OX/GA, MC38 tumor-bearing C57BL/6 mice showed 100% tumor eradication in 2 weeks (Fig. 3A). In comparison, OX+GA only slightly inhibited tumor growth with a TGI value of 25.0%. OX-NCP moderately inhibited tumor growth with a TGI of 53.8%. Although GA-NCP showed a TGI of 82.4%, the tumors regrew rapidly after cessation of the treatment. Five doses of OX/GA did not result in obvious weight loss or abnormalities in major organs (figs. S7C and S8).

We also analyzed mouse survival based on prespecified tumor sizes as the endpoint (Fig. 3B). OX+GA only increased median survival by 2 days from 27.5 days for the PBS group. OX-NCP and GA-NCP moderately prolonged the median survival to 33 and 39 days, respectively. All the mice in the OX/GA group were tumor-free and healthy up to day 60 when the mice reached the monitoring endpoint. At 60 days after MC38 tumor inoculation, all the mice from the OX/GA group were challenged with fresh MC38 cells (Fig. 3, C and D). None of the six mice had relapse, indicating immune memory effects against MC38 cells in the cured mice. Hematoxylin and eosin (H&E) staining of OX/GA-treated tumors showed an abundance of dead cells with nuclear chromatin pyknosis and cytoplasm disappearance (Fig. 3E and fig. S9). Terminal deoxynucleotidyl transferase–mediated deoxyuridine triphosphate nick end labeling (TUNEL) staining further confirmed high percentages of apoptotic cells with highly fragmented DNA in OX/GA-treated tumors.

In the CT26 model, OX/GA also showed potent anticancer activity with a TGI of 95.3% and suppressed the tumors after five doses with steady body weight (Fig. 3F and fig. S7D). In comparison, OX+GA, OX-NCP, and GA-NCP demonstrated moderate TGI values of 62.6, 71.3, and 30.1%, respectively. OX/GA significantly prolonged median survival to 40.5 days from 22 days for PBS (Fig. 3G). OX+GA, OX-NCP, and GA-NCP moderately extended the median survival to 24, 29, and 30.5 days, respectively.

We evaluated the antitumor efficacy of OX/GA in *Tek*^Δ*STING*^, *Tmem173^−/−^*, and WT mice (fig. S7E). In comparison to the robust tumor eradication observed in WT mice, OX/GA exhibited moderate and minimal antitumor effects in MC38 tumor-bearing *Tek*^Δ*STING*^ mice with a TGI value of 61.9% and *Tmem173^−/−^* mice with a TGI value of 24.0%. These findings support the role of tumor vasculature disruption on the antitumor efficacy of OX/GA.

The antitumor efficacy of OX/GA was further evaluated in the C57BL/6-Tg(TRAMP)8247Ng/J (TRAMP) spontaneous prostate cancer model (*39*). Male TRAMP mice start to develop spontaneous autochthonous prostate tumors at ~10 weeks of age. They begin to form metastases in the peripheral lymph nodes, lungs, and, occasionally, other organs at about 12 weeks (*40*). Thus, this genetically engineered mouse model (GEMM) of prostate cancer better recapitulates human tumors than subcutaneous tumor models. We verified TRAMP mice by genotyping and used 24-week-old male TRAMP mice for antitumor efficacy studies. After treatment with PBS, OX+GA, GA-NCP, or OX/GA Q3D for 10 doses, the prostates and seminal vesicles of TRAMP mice were harvested, weighed, and photographed at 30 days after the first administration (Fig. 3H). Ratios of genitourinary tract weight to body weight were used to evaluate antitumor efficacy. OX/GA reduced tumor burden by 99.9% compared to PBS control (Fig. 3I). Furthermore, OX/GA-treated TRAMP mice had a healthy appearance and showed similar genitourinary tract weights to WT B6 mice (Fig. 3J). However, OX+GA only reduced tumor burden by 2.5%, while GA-NCP reduced tumor burden by 62.2%. This result demonstrates the potency of OX/GA in the treatment of a clinically relevant spontaneous tumor model that closely mimics human prostate cancer.

We also evaluated the antitumor efficacy of OX/GA in an orthotopic pancreatic cancer model (Fig. 3K). The orthotopic pancreatic cancer model was developed by implanting Panc02 tumor cells into the pancreas of C57BL/6 mice and monitored by ultrasound imaging. When tumors reached >0.5 cm in any dimension, tumor-bearing mice were intravenously injected with PBS, OX+GA, or OX/GA Q3D for 10 doses. The mice in the PBS group had to be euthanized due to tumor burden after four doses. OX+GA slightly inhibited tumor growth with a TGI of 32.6%. OX/GA strongly suppressed tumor growth with a TGI of 98.0% and a remarkable cure rate of 60% (Fig. 3, L to N). OX+GA slightly prolonged the median survival to 37 days from 26 days for PBS. OX/GA greatly extended the median survival to >60 days. Thus, OX/GA exhibited potent antitumor effects in the immunologically cold orthotopic pancreatic tumor model (*41*).

We further assessed the antitumor effects of OX/GA using a less immunogenic, orthotopic, and metastatic 4T1 triple-negative breast cancer (TNBC) model (Fig. 3O). The 4T1 model was established by injecting 4T1 cells into the mammary glands of female BABL/c mice. Once the tumor volume reached ~80 mm^3^, the mice received the treatments as described earlier. OX/GA exhibited robust antitumor activity with a TGI value of 98.1%. In comparison, OX+GA, OX-NCP, and GA-NCP displayed moderate antitumor effects with TGI values of 24.3, 50.6, and 71.1%, respectively (Fig. 3P). OX/GA significantly extended the median survival of mice to 51.5 days from 25 days for the PBS group. In comparison, OX+GA, OX-NCP, and GA-NCP moderately increased median survival to 29, 38.5, and 43 days, respectively (Fig. 3Q). Analysis of lung tissues using H&E staining at the endpoint of the PBS group revealed a substantial reduction in metastatic clones in the lung sections of the mice treated with OX/GA compared to other groups (Fig. 3R). OX/GA demonstrated strong antimetastatic effects with 0% lung metastasis, while OX+GA, OX-NCP, and GA-NCP showed moderate lung metastasis rates of 23.5, 20.7, and 5.7%, respectively (fig. S10). These findings demonstrate the effective inhibition of distant metastasis of TNBC by OX/GA, resulting in prolonged survival of mice.

### OX/GA enhances TAA presentation

**Fig. 4.**
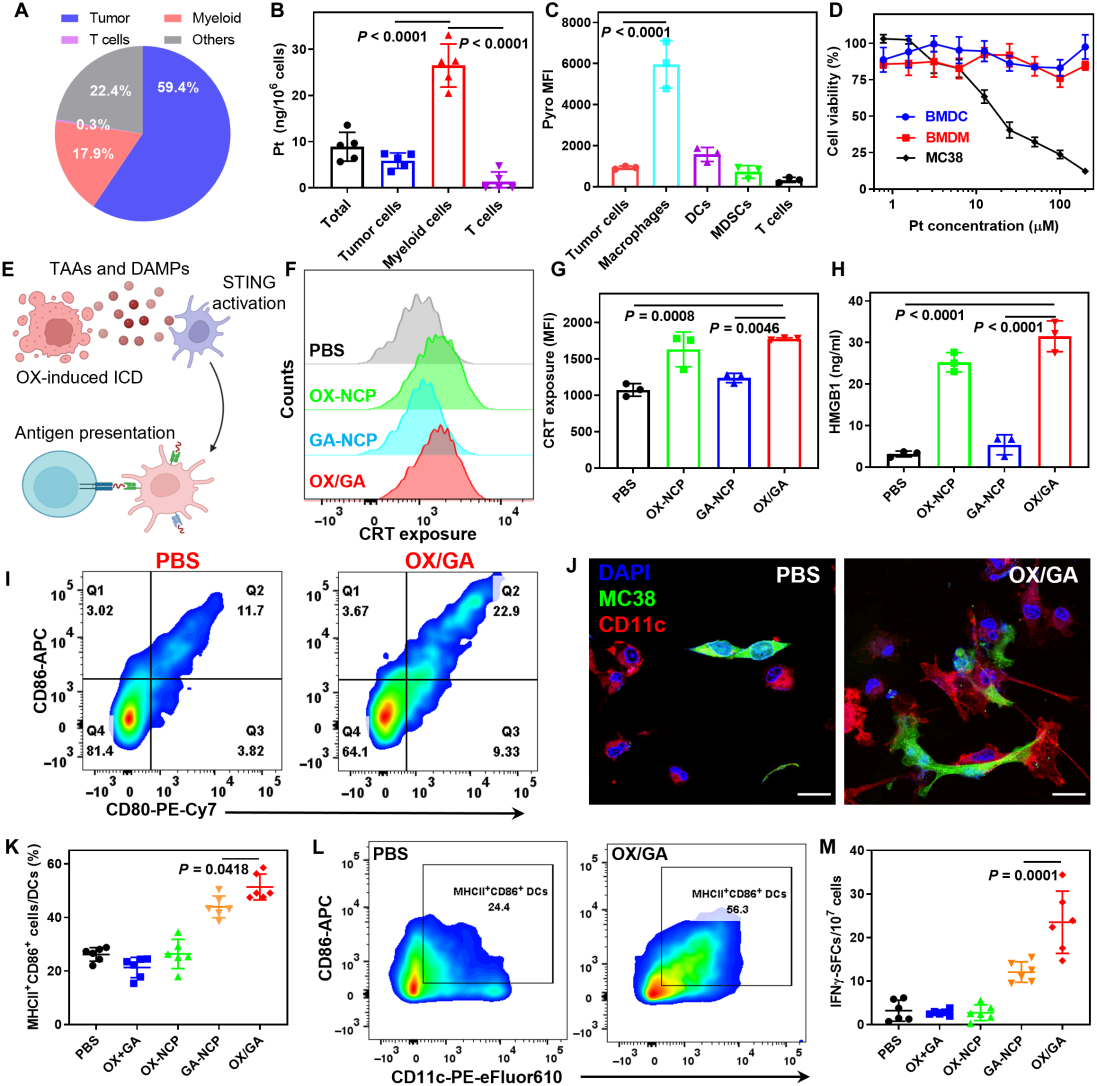
OX/GA enhances antigen presentation. (**A** and **B**) Pt distributions (A) and levels (B) in different subsets of cells in MC38 tumors 24 hours after intravenous injection of OX/GA (*n* = 5). (**C**) Pyro MFIs in different subsets of cells in MC38 tumors 24 hours after intravenous injection of OX/GA-pyro (*n* = 3). (**D**) MTS assays of OX/GA in MC38 cells, BMDCs, and BMDMs (*n* = 3). (**E**) Schematic of OX/GA-mediated STING activation. (**F** and **G**) Flow cytometry analysis of histogram (F) and MFI (G) of CRT exposure after 24-hour incubation with drugs at a dose of 15 μM of OX and/or 1 μM of GA (*n* = 3). (**H**) ELISA for HMGB1 release after 24-hour incubation with drugs at a dose of 15 μM of OX and/or 1 μM of GA (*n* = 3). (**I**) In vitro DC maturation induced by OX/GA-treated MC38 cells. (**J**) CLSM images showing phagocytosis of OX/GA-treated MC38 cells (CFSE; green) by BMDCs (CD11c; red). Scale bars, 20 μm. (**K** and **L**) In vivo DC maturation in TDLNs from MC38 tumor-bearing C57BL/6 mice 4 days after the treatment (*n* = 6). (**M**) ELISpot assay detecting MC38 tumor-specific IFN-γ secreting splenocytes 12 days after the first treatment (*n* = 6). Data in (B) to (D), (G), (H), (K), and (M) are presented as means ± SD. The data were analyzed by one-way ANOVA with Tukey’s multiple comparisons tests. The scheme in (E) was created with BioRender.com.

We investigated the mechanisms for the potent antitumor effect of OX/GA. First, we evaluated the cellular distribution of OX/GA in MC38 tumors 24 hours after intravenous injection by ICP-MS analysis of Pt. Tumor cells and myeloid cells uptook most OX/GA at 59.4 and 17.9%, respectively (Fig. 4A and fig. S11). T cells only uptook 0.3% of OX/GA. The average Pt level in each myeloid cell was 4.5-fold higher than that in each tumor cell, suggesting preferential uptake of OX/GA by myeloid cells (Fig. 4B). The distribution of OX/GA ensures efficient chemotherapeutic effects to kill cancer cells and sufficient delivery of GA to immune cells for STING activation. We further analyzed the uptake of OX/GA-pyro by different myeloid cell populations (Fig. 4C and fig. S11). Flow cytometry analyses showed that macrophages had a higher fluorescence signal with 6.4-fold mean fluorescence intensity (MFI) of pyro over tumor cells. DCs showed 1.7-fold higher pyro MFI than tumor cells. In addition, we compared the uptake of OX/GA-pyro in ECs to other cell types (fig. S12). ECs exhibited the highest fluorescence signal, with a 1.4-fold higher MFI of pyro than macrophages and a remarkable 7.4-fold increase in MFI over tumor cells. These results provide further evidence for the substantial uptake of OX/GA by ECs within the tumor and support tumor vasculature disruption via EC STING activation.

We next determined the cytotoxicity of OX/GA in different cell lines. OX/GA was toxic to MC38 tumor cells with an OX median inhibitory concentration (IC_50_) of 14.6 μM but nontoxic to macrophages and DCs at concentrations of up to 200 μM (Fig. 4D). On the basis of the cellular uptake and toxicity results, we hypothesized that OX/GA served as a bifunctional nanoplatform that efficiently kills tumor cells to release TAAs and activates myeloid cells (Fig. 4E). OX is among the few chemotherapeutics that efficiently cause ICD (*42*–*45*). We previously showed that OX-loaded NCP induced ICD of MC38 cells and generated damage-associated molecular patterns (DAMPs) (*35*, *46*). Calreticulin (CRT) staining demonstrated the translocation of CRT onto the surface of MC38 cells upon treatment with OX-NCP and OX/GA (Fig. 4, F and G). OX/GA significantly enhanced high mobility group box 1 protein (HMGB1) release in MC38 cells by 10-fold compared to the PBS control (Fig. 4H). These findings provide compelling evidence for ICD induction by OX/GA treatment to release TAAs and DAMPs.

A Transwell system was used for the coculturing of BMDCs with pretreated MC38 cells to investigate the role of OX/GA in antigen presentation. Flow cytometry analyses of CD80/CD86 in DCs revealed DC activation and maturation by DAMPs (Fig. 4I and fig. S13). MC38 cells treated with OX/GA increased the populations of mature DCs (mDCs) to 22.9% from 11.7% for PBS control. The direct addition of OX/GA to DCs increased the mDC population to 17.5% due to directly stimulating the STING pathway in DCs. We further evaluated the phagocytosis by coincubating BMDCs and OX/GA-treated MC38 cells. Confocal laser scanning microscopy (CLSM) studies showed that OX/GA treatment induced apoptosis of MC38 cells and substantially increased phagocytosis by BMDCs (Fig. 4J and fig. S14).

We also evaluated the effect of OX/GA on antigen presentation in vivo. Tumor-draining lymph nodes (TDLNs) of MC38 tumor-bearing C57BL/6 mice treated with PBS, OX+GA, OX-NCP, GA-NCP, or OX/GA were collected and analyzed for MHCII^+^CD86^+^ expressions in DCs (CD45^+^CD11b^+^CD11c^+^) 4 days after treatment. OX+GA or OX-NCP treatment did not increase MHCII^+^CD86^+^ expression over PBS control (26.2%). GA-NCP increased MHCII^+^CD86^+^ population to 41.0%. OX/GA significantly enhanced MHCII^+^CD86^+^ expression by two- and 1.3-fold compared to PBS and GA-NCP, respectively (Fig. 4, K and L, and fig. S15).

To further investigate TAA presentation by OX/GA, we performed IFN-γ enzyme-linked immunospot (ELISpot) assay with splenocytes from MC38 tumor-bearing C57BL/6 mice 12 days after the first treatment. GA-NCP treatment significantly increased IFN-γ secreting spot-forming cells (SFCs) to 1.21 per million cells from 0.32 SFCs per million cells for PBS control. OX/GA further enhanced adaptive immunity to afford 2.35 SFCs per million cells (Fig. 4M). Together, OX/GA treatment not only releases TAAs and DAMPs by causing ICD of tumor cells but also enhances TAA presentation by stimulating myeloid cells, leading to successful adaptive immune response to suppress tumors in multiple cancer models.

### OX/GA stimulates innate immune responses and repolarizes TAMs

**Fig. 5.**
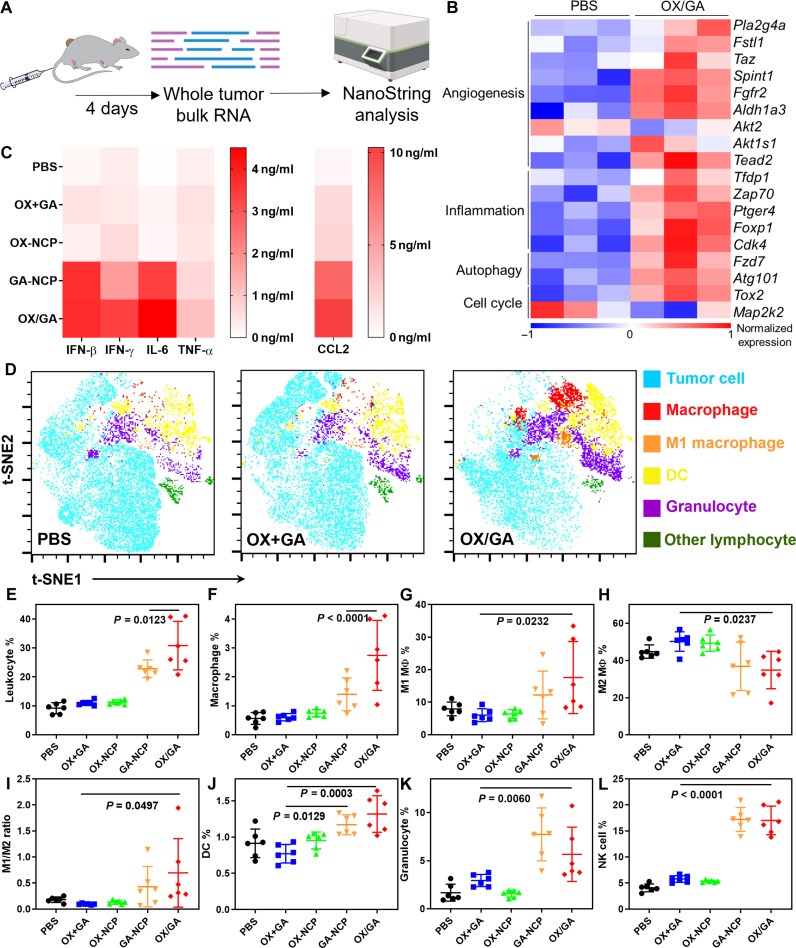
OX/GA stimulates innate immune responses and induces inflammatory TME. (**A** and **B**) Schematic showing NanoString analysis (A) and heatmaps showing differential expression of selected genes (B) in bulk tumors 4 days after OX/GA or PBS treatment. (**C**) Heatmaps of proinflammatory cytokines levels in the tumors at 24 hours after indicated treatments. C57BL/6 mice were intravenously injected with PBS, OX+GA, OX-NCP, GA-NCP, or OX/GA at 3.0 mg OX /kg and/or 0.36 mg GA/kg (*n* = 3). (**D** to **L**) Immune cell infiltration in tumors on day 4 after treatment quantified by FACS. (D) Representative t-SNE analysis for MC38 tumors treated with PBS, OX+GA, and OX/GA. In FlowJo software, Exact (vantage point tree) *k*-nearest neighbors algorithm and Barnes-Hut gradient algorithm were used in the t-SNE analysis. [(E) to (I)] Immune cell subpopulations in MC38 tumors. The subpopulations were defined as follows: (E) leukocytes as CD45^+^; (F) macrophages as CD45^+^CD11b^+^F4/80^+^; [(G) to (I)] M1-like macrophages (MΦ) as CD45^+^CD11b^+^F4/80^+^CD86^+^ and M2-like macrophages as CD45^+^CD11b^+^F4/80^+^CD206^+^; (J) DCs as CD45^+^CD11b^+^CD11c^+^ MHCII^+^; (K) granulocytes/MDSC as CD45^+^CD11b^+^GR-1^+^F4/80^−^; and (L) NK cells as CD45^+^CD3e^−^NK1.1^+^ (*n* = 6). Data are presented as means ± SD. The data in (E) to (H) and (J) to (L) were analyzed by one-way ANOVA with Tukey’s multiple comparisons tests. The data in (I) were analyzed by Student’s two-tailed *t* test. The scheme in (A) was created with BioRender.com.

We excised the MC38 tumors after PBS or OX/GA treatment and extracted total RNAs for quantification of differential gene expressions with the NanoString nCounter Tumor Signaling 360 Panel (Fig. 5A). Four days after the first treatment, OX/GA-treated tumors showed up-regulation of angiogenesis genes, likely as a compensatory response to the severe disruption of tumor vasculatures (Fig. 5B) (*47*, *48*). OX/GA also up-regulated genes associated with inflammation and autophagy, indicating that OX/GA induced a strong inflammatory environment in the tumors (Fig. 5B). Between PBS and OX/GA groups, 23 related genes passed the threshold of ≥1.5-fold expression changes and adjacent *P* values (*P*_adj_) of <0.05 (figs. S16 to S18). In contrast, OX+GA and GA-NCP exhibited fewer alterations with six and nine genes passing the threshold, respectively. Gene set analysis revealed that OX/GA showed higher scores for angiogenesis and innate immune response than free OX+GA and GA-NCP (fig. S18). Furthermore, OX/GA showed alterations of genes associated with cell cycle and apoptosis, likely in response to OX-induced cell cycle arrest and ICD.

To confirm the inflammatory TME, we analyzed proinflammatory cytokines in the tumors 24 hours after treatment (Fig. 5C and fig. S19). OX/GA treatment significantly stimulated the secretion of proinflammatory cytokines in the tumors, with 24.1-, 10.2-, 563.0-, 2.5-, and 19.3-fold higher IFN-β, IFN-γ, interleukin-6 IL-6, TNF-α, and chemokine ligand 2 (CCL2), respectively, over PBS control. Treatment with GA-NCP showed similarly high secretion of proinflammatory cytokines in the tumors. These results are consistent with the generation of highly inflammatory TMEs by OX/GA via synergistic STING activation by GA and the release of DAMPs by OX, leading to enhanced antigen presentation.

We profiled leukocytes in the tumors 4 days after the first treatment by flow cytometry (Fig. 5, D to L, and fig. S20). The t-distributed stochastic neighbor embedding (t-SNE) analysis revealed a notable decrease in tumor cells and a substantial increase in tumor-infiltrated innate immune cells, specifically DCs, macrophages (including M1-like macrophages), and granulocytes in OX/GA-treated tumors (Fig. 5D). OX+GA did not exhibit population difference from PBS control. The levels of total infiltrating CD45^+^ leukocytes were similar among PBS, OX plus GA, and OX-NCP groups (9.1 to 11.3%), but GA-NCP and OX/GA groups showed two- to threefold higher CD45^+^ leukocyte percentages at 22.8 and 30.8%, respectively (Fig. 5E and fig. S21). OX/GA enhanced infiltration of CD11b^+^F4/80^+^ macrophages in the tumors, with a 4.9-fold increase over the PBS group (2.77% for OX/GA versus 0.56% for PBS; Fig. 5F). Neither OX+GA nor OX-NCP enhanced macrophage infiltration, while GA-NCP moderately increased infiltration of macrophages (1.40%) by 2.5-fold over PBS control.

While most of these TAMs in the tumors were anti-inflammatory M2-like phenotype (F4/80^+^CD206^+^) in the PBS group, OX/GA treatment induced notable macrophage repolarization to M1-like phenotype (F4/80^+^CD86^+^) with a 3.7-fold higher M1/M2 ratio than that of the PBS group (0.66 for OX/GA versus 0.18 for PBS; Fig. 5, G to I, and fig. S22), which was consistent with t-SNE analysis (Fig. 5D). Neither OX+GA nor OX-NCP treatment repolarized macrophages, but GA-NCP induced moderate M1-like macrophage repolarization with an M1/M2 ratio of 0.43. OX/GA and GA-NCP increased infiltration of CD11c^+^MHCII^+^ DCs in the tumors by 1.44- and 1.28-fold over PBS control, respectively (Fig. 5J). OX/GA and GA-NCP treatments also increased Gr-1^+^F4/80^−^ granulocytes in the tumors (Fig. 5K). OX/GA and GA-NCP treatment significantly increased the infiltration of natural killer (NK) cells in the tumors by ~4.2-fold over PBS control (17.0, 17.3, and 4.1% for OX/GA, GA-NCP, and PBS, respectively; Fig. 5L). These observations suggest that OX/GA stimulates a strong innate immune response by recruiting innate immune cells and repolarizing anti-inflammatory subpopulations into an antitumor state.

### OX/GA activates adaptive immunity and enhances ICB

**Fig. 6.**
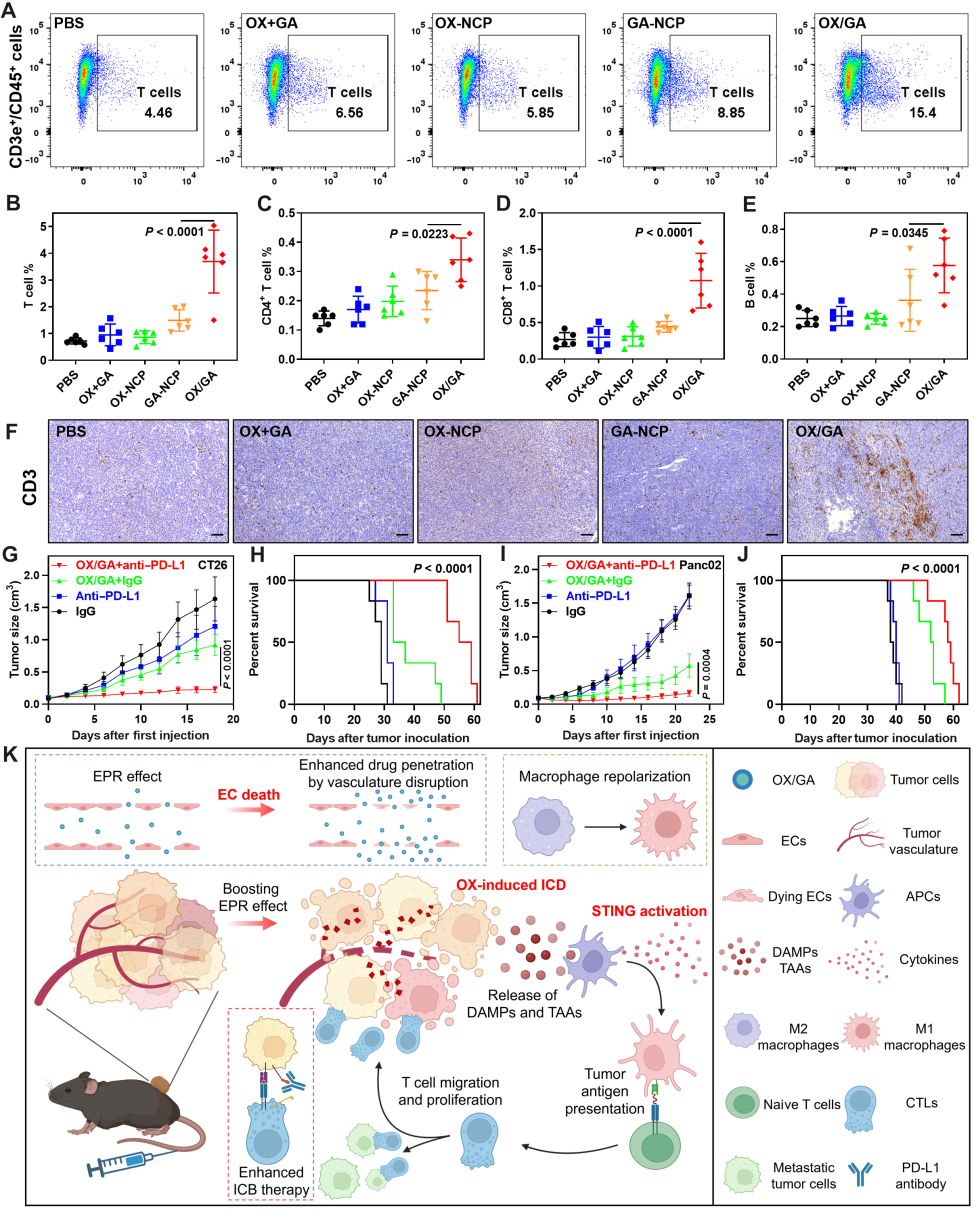
OX/GA activates adaptive immunity and enhances ICB. (**A**) Representative flow cytometry plots of T cells (CD45^+^CD3e^+^) among leukocytes (CD45^+^) from MC38 tumor-bearing C57BL/6 mice 12 days after treatment. (**B** to **E**) Adaptive immune cell profiles in tumors on day 12 after the first dose (for a total of four doses) quantified by FACS. The subpopulations were defined as follows: (B) T cells as CD45^+^CD3e^+^; (C) CD4^+^ T cells as CD45^+^CD3e^+^CD4^+^; (D) CD8^+^ T as CD45^+^CD3e^+^CD8^+^; and (E) B cells as CD45^+^ B220^+^ (*n* = 6). (**F**) CD3 IHC slides of MC38 tumor-bearing C57BL/6 mice 12 days after the first dose. (**G** and **H**) Tumor growth curves and survival curves of CT26 tumor-bearing BALB/c mice after indicated treatments (*n* = 6). Mice were intravenously injected with OX/GA at equivalents of 2.0 mg OX/kg and 0.24 mg GA/kg and intraperitoneally injected with 100 μg of IgG or anti–PD-L1 antibodies Q3D for five doses in total. (**I** and **J**) Tumor growth curves and survival curves of Panc02 tumor-bearing C57BL/6 mice after indicated treatments (*n* = 6). Mice were intravenously injected with OX/GA at equivalents of 3.0 mg OX/kg and 0.36 mg GA/kg and intraperitoneally injected with 100 μg of IgG or anti–PD-L1 antibodies Q3D for five doses in total (*n* = 6). (**K**) Schematic depicting mechanism of action of OX/GA. OX/GA causes potent vasculature disruption, allowing enhanced drug penetration into tumor tissue; in cells, OX/GA induces ICD and activates STING, inducing the release of DAMPs and TAAs and repolarizing macrophages and enabling immune priming of cold tumors in combination with PD-L1 antibody. Data in (B) to (E), (G), and (I) are presented as means ± SD. The data were analyzed by one-way ANOVA with Tukey’s multiple comparisons tests. A log-rank (Mantel-Cox) test was used for the statistical analysis of survival curves. The scheme in (K) was created with BioRender.com.

We further evaluated adaptive antitumor immunity induced by OX/GA. The infiltrated immune cells in MC38 tumors were analyzed by flow cytometry 12 days after the first treatment (for a total of four doses; fig. S23). The population of T cells markedly increased in the tumors treated with OX/GA (3.69%), which was 5.1-fold higher than that of PBS control (Fig. 6, A and B). GA-NCP moderately enhanced T cell infiltration by 2.1-fold over PBS control, while neither OX+GA nor OX-NCP changed the T cell percentages in the tumors. OX/GA increased CD4^+^ T cells by 2.4-fold over PBS (0.34% versus 0.14%, Fig. 6C) and increased CD8^+^ T cells by fourfold over PBS (1.07% versus 0.27%, Fig. 6D). OX/GA also increased the tumor infiltration of B cells by 2.3-fold over PBS (Fig. 6E and fig. S24). Tumor IHC staining of the CD3 marker revealed obviously increased infiltration of T cells in the OX/GA group over other groups (Fig. 6F). We also analyzed IFN-γ^+^CD8^+^ T cells and granzyme B^+^CD8^+^ T cells to demonstrate the cytotoxic capabilities of these CD8^+^ T cells (fig. S25). GA-NCP treatment resulted in a moderate increase in both IFN-γ^+^CD8^+^ T cells and granzyme B^+^CD8^+^ T cells by 6.7- and 2.3-fold, respectively, compared to the PBS control, whereas treatments with OX+GA or OX-NCP did not affect these cell populations in the tumors. Notably, OX/GA treatment significantly increased the levels of IFN-γ^+^CD8^+^ T cells and granzyme B^+^CD8^+^ T cells by 54.0- and 7.2-fold over PBS, respectively. These results demonstrate that OX/GA elicits strong antitumor immunity by engaging the adaptive immune system via the synergistic actions of OX-induced ICD and GA-induced STING activation.

ICB has had tremendous clinical success in some immunogenic tumors such as melanoma and non–small cell lung cancer but has not provided survival benefits to patients with non–T cell inflamed or cold tumors (*41*). Because OX/GA activated the immune infiltration and created a T cell inflamed TME, we hypothesized that OX/GA could overcome resistance to ICB in cold tumors. We first tested this hypothesis in the CT26 tumor model, which is less immunogenic (Fig. 6, G and H) (*49*, *50*). At a lower (2/3) dose, OX/GA plus immunoglobulin G (IgG) isotype control showed a moderate antitumor effect with a TGI of 43.8% after five doses on a Q3D schedule. The intraperitoneal administration of anti–PD-L1 antibody for five doses only showed a modest TGI of 26.1%. In contrast, OX/GA plus anti–PD-L1 exhibited strong TGI with a TGI of 85.9%. OX/GA plus anti–PD-L1 extended the median survival of mice to 57 days from 29 days for PBS, whereas anti–PD-L1 and OX/GA plus IgG slightly extended mouse median survival to 31 and 35 days, respectively.

We next tested the OX/GA plus anti–PD-L1 combination on the cold Panc02 tumor model, which did not respond to anti–PD-L1 treatment (Fig. 6, I and J). Panc02 tumors moderately responded to OX/GA plus IgG treatment with a TGI of 64.2%, which is likely due to the lower sensitivity of Panc02 cells (IC_50_ = 50.8 μM) to OX/GA than MC38 cells (IC_50_ = 14.6 μM) (fig. S26). OX/GA plus ant–PD-L1 treatment effectively inhibited the growth of Panc02 tumors with a TGI of 90.0%. OX/GA plus anti–PD-L1 treatment significantly extended the median survival of mice to 58.8 days from 38.5 days for PBS. In comparison, OX/GA plus IgG extended the median survival of mice to 52.5 days but anti–PD-L1 alone did not improve survival. These results indicate that the combination of OX/GA with ICB is effective against immunologically cold tumors, which respond poorly to ICB alone.

## DISCUSSION

Nanotherapeutics have been extensively explored for cancer therapy in the past three decades, largely motivated by the postulation of the EPR effect in tumors (*51*). It is hypothesized that the leaky neovasculature and the ineffective lymphatic drainage in tumors can increase tumor deposition of drug payloads in long-circulating nanotherapeutics (*17*, *52*–*54*). Despite extensive efforts, only a few nanotherapeutics have been approved by the U.S. Food and Drug Administration for cancer treatment (*55*–*57*). EPR-mediated nanoparticle accumulation in tumors is limited by the heterogeneity of pathophysiological parameters in different tumors, including vascular permeability, vascular maturation at the vessel level (*22*), extracellular matrix (*23*), and interstitial fluid pressure (*24*). Existing nanotherapeutics cannot effectively overcome these barriers to deposit sufficient payloads in tumors and elicit potent antitumor effects without causing general toxicity. For example, although Doxil (liposomal doxorubicin) has a higher intratumoral accumulation than free doxorubicin, the particles are restricted within perivascular regions without extravasation to the bulk of the tumors, resulting in a narrow therapeutic index.

Systemic delivery of CDN-based STING agonists is limited by their poor PK and instability in plasma (*34*, *58*–*60*). Insufficient TAA presentation and asynchronous immune activation have led to disappointing clinical readouts. However, by combining with other treatment modalities using efficient delivery systems, CDN STING agonists have shown the potential to augment the immunotherapeutic effects (*61*). Here, by coordinatively immobilizing GA in the zinc-phosphate core, NCP protected GA from enzymatic degradation and prolonged GA blood circulation by 56.3-fold. Furthermore, bifunctional OX/GA particles markedly boost the EPR effects via endothelial STING-mediated tumor vascular disruption (Fig. 6K). At a nontoxic dose, OX/GA activated STING in tumor ECs to disrupt tumor vasculature and enhance therapeutic uptake and permeabilization, resulting in a 4.9-fold increase in the AUC for OX over OX-NCP. OX/GA preferentially induced ICD of cancer cells without killing immune cells, leading to the enhanced presentation of TAAs for adaptive immune responses. Therefore, NCP offers a strategy to overcome the limitations of the EPR effect and thus improve tumor-targeting of chemotherapeutics and STING agonists by stabilizing the drugs, improving their PK, and preferentially targeting antigen-presenting cells (APCs).

Except for the use of male mice in the GEMM TRAMP model, this study exclusively used female mice. This presents a potential limitation, particularly concerning the generalizability of our findings. The decision on the use of female mice in this study was based on variable control and the required use of female mice to establish the 4T1 model. It is known that immune responses can differ between sexes, potentially influencing both the natural course of diseases and the efficacy of therapeutic interventions. Our future studies will include both male and female individuals to provide a more comprehensive understanding of the immune responses and therapeutic effects in a sex-inclusive manner.

In summary, OX/GA functions as a tumor-targeted nanocarrier for a STING agonist and a potent ICD-inducing chemotherapeutic. First, the encapsulation of hydrophilic GA and OX-bp in the NCP core protects the drugs from enzymatic degradation and enhances their PK upon systemic administration. Second, OX/GA induces strong endothelial STING activation for tumor vasculature disruption. The resulting leakier tumor vasculatures further enhance tumor accumulation of OX and GA to overcome the limitations of EPR-mediated nanoparticle accumulation in tumors. Third, OX potently induces ICD to release TAAs and DAMPs without killing immune cells, and preferential uptake of OX/GA by APCs activate STING in the TME to enhance the presentation of TAAs by DCs. Fourth, OX/GA stimulates the release of proinflammatory cytokines in the tumors to enhance innate and adaptive immune responses and facilitates the infiltration of immune cells to suppress tumor growth. Last, the combination of OX/GA with anti–PD-L1 reverses immunosuppression to a “hot” TME and reinvigorates T cells for cancer cell killing. Thus, our work shows that NCPs provide an excellent platform for the development of immunostimulatory agents and the integration of multiple therapeutic regimens, including chemotherapy and immunotherapy, for effective cancer therapy.

## MATERIALS AND METHODS

### Materials, cell lines, and animals

All starting materials were purchased from Sigma-Aldrich and Fisher (United States), unless otherwise noted, and used without further purification. Mammalian (noncanonical) CDN, cyclic [G(2′,5′) pA(3′,5′)p] (2′3′-GA) was purchased from InvivoGen. DOPA, DOPC, cholesterol, and DSPE-PEG_2000_ were purchased from Avanti Polar Lipids (United States).

Murine colorectal carcinoma cells MC38, CT26, breast carcinoma cell 4T1, pancreatic carcinoma cell Panc02, and primary HUVECs were purchased from the American Type Culture Collection (Rockville, MD, United States). Human THP1-Dual KO-MyD88 monocytes were purchased from InvivoGen and cultured according to the vendor’s protocol. The cells were cultured in Dulbecco’s modified Eagle’s medium or RPMI 1640 medium (Gibco, Grand Island, NY, United States), supplemented with 10% heat-inactivated fetal bovine serum (HI-FBS; 56°C water bath for 30 min; VWR, United States), penicillin G sodium (100 U/ml), and streptomycin sulfate (100 g/ml) in a humidified atmosphere containing 5% CO_2_ at 37°C. HUVECs were maintained in vascular cell basal medium, supplemented with Endothelial Cell Growth Kit-BBE and Penicillin/Streptomycin/Amphotericin B Solution. Exponentially growing cultures were kept in the incubator at 37°C with an atmosphere of 5% CO_2_ and 90% relative humidity. The subculture of ECs was mandatory when cultures reached ~80% confluence.

C57BL/6 mice, BALB/c mice (6 to 8 weeks), and SD/CD female rats (6 weeks, 160 to 200 g) were obtained from Charles River Laboratories Inc. (United States). *Tmem173^−/−^*, *STING^flox^*, and *Tek^Cre^* mice were purchased from the Jackson Laboratory. *Tek*^ΔSTING^ mice were bred from the *STING^flox^* and *Tek^Cre^* mice. The study protocol was reviewed and approved by the Institutional Animal Care and Use Committee at the University of Chicago.

### Preparation and characterization of OX/GA

Aqueous solutions of OX-bp (30 mg, 150 mg/ml) and GA (2 mg, 10 mg/ml) were added to 5 ml of 0.3 M Triton X-100/1.5 M 1-hexanol in cyclohexane and stirred vigorously for 15 min in the presence of DOPA (30 mg, 200 mg/ml in CHCl_3_). An aqueous solution of Zn(NO_3_)_2_ (60 mg, 600 mg/ml) was added to 5 ml of 0.3 M Triton X-100/1.5 M 1-hexanol in cyclohexane and stirred vigorously for 5 min. The Zn(NO_3_)_2_-containing microemulsion was added dropwise to the OX- and GA-containing microemulsion and stirred vigorously for 30 min at room temperature. After the addition of 10 ml of ethanol, OX-bare was obtained by centrifugation at 11,628*g*. The resulting pellet was washed twice with THF/ethanol and lastly redispersed in THF. The loadings of OX in the particles were determined by ICP-MS (Agilent 7700×, Agilent Technologies, United States) after digestion with nitric acid.

OX/GA was prepared by adding a THF solution (80 μl) of DOPC, cholesterol, DSPE-PEG_2000_ (2:2:1), and OX/GA-bare to 500 μl of 30% (v/v) ethanol/water at room temperature. The mixture was stirred at 1700 rpm for 1 min. THF and ethanol were completely evaporated at 50°C by blowing nitrogen gas, and the solution was allowed to cool to room temperature. The particle size and zeta potential were determined by dynamic light scattering (DLS) using a Zetasizer (Nano ZS, Malvern, United Kingdom). Transmission electron microscopy (TEM) (Tecnai Spirit, FEI, United States) was used to observe particle morphology. To determine GA loading, OX/GA was digested in 0.01 M HCl solution for 10 min. The mixture was vortexed and centrifuged at 10,000*g* for 5 min, and the supernatant was determined by liquid chromatography–mass spectrometry (Agilent 6540, Agilent Technologies, United States). Fluorescent control particles OX-pyro and OX/GA-pyro were similarly prepared and characterized.

### BMDCs and BMDMs

Six- to 8-week-old female C57BL/c mice were euthanized, and bone marrow cells were flushed out from the femur and tibia using insulin syringes with RPMI 1640. Red blood cells were lysed by sterile ACK buffer (Corning), and the rest of the cells were cultured in RPMI 1640 full medium + recombinant mouse granulocyte-macrophage colony-stimulating factor (GM-CSF) (20 ng/ml; R&D Systems) + recombinant murine interleukin-4 (IL-4) (10 ng/ml; PeproTech). On day 4, the entire medium was discarded and replaced by a fresh and warm medium with GM-CSF (20 ng/ml) and IL-4 (10 ng/ml). On day 6, the semisuspended and loosely attached cells were collected by gently pipetting and the medium suspension was collected as BMDCs. The adherent cells were gently scraped off by cell scrapers as bone marrow–derived macrophages (BMDMs). The purity of the cells was examined by flow cytometry with CD11c-PE-eFluor610 (N418) and F4/80-PerCP/Cy5.5 (BM8) antibodies, respectively.

### STING activation in vitro and ex vivo

THP1-Dual KO-MyD88 reporter cells were used to quantify STING activation of GA and OX/GA in vitro. The cells were seeded in 96-well plates at a density of 10^5^ cells/ml. GA or OX/GA was added, and the cells were incubated for 24 hours. The stimulation of the IRF pathway was quantified by QUANTI-Luc (InvivoGen) assay on a Synergy HTX plate reader.

Ex vivo study was carried out by measuring IFN-β secreted by BMDCs. A total of 5 × 10^5^ cells were seeded in six-well plates. PBS, OX, GA, OX+GA, OX-NCP, GA-NCP, or OX/GA at an equivalent concentration of 15 μM OX and/or 1 μM GA was added to the cells. After 24-hour incubation, the medium was collected and IFN-β was quantified by LumiKine Xpress mIFN-β 2.0 (InvivoGen) mouse enzyme-linked immunosorbent assay (ELISA) kit (Invitrogen).

### DC maturation

DC activation study was carried out by evaluating the expression of CD80 and CD86 on BMDCs. A total of 5 × 10^5^ cells were seeded in six-well plates. PBS, OX, GA, OX+GA, OX-NCP, GA-NCP, or OX/GA at an equivalent concentration of 15 μM OX and/or 1 μM GA was added to the cells. After 24-hour incubation, the cells were transferred from the plate to Eppendorf Tubes and incubated with anti-CD16/32 (clone: 93; eBioscience, 1:100) to reduce nonspecific binding to Fc receptors (FcRs). The cells were further stained with the APC anti-mouse CD86 antibody (clone: GL1; eBioscience, 1:100) and PE-Cy7 anti-mouse CD80 antibody (clone: 16-10A1; eBioscience, 1:100) on ice for 0.5 hours. After washing with PBS, the cells were resuspended with fluorescence-activated cell sorting (FACS) buffer and analyzed on an LSRFortessa 4-15 flow cytometer.

In the coculture assay, MC38 cells (1 × 10^5^ cells per well) and BMDCs (5 × 10^5^ cells per well) were seeded in the upper and lower compartments of Transwells, respectively. MC38 cells were incubated with different treatments at an equivalent concentration of 15 μM OX and/or 1 μM GA for 24 hours and then cocultured with BMDCs for another 24 hours. Then, BMDCs were transferred from the plate to Eppendorf Tubes and incubated with anti-CD16/32 (clone: 93; eBioscience, 1:100) to reduce nonspecific binding to FcRs. The cells were further stained with the APC anti-mouse CD86 antibody (clone: GL1; eBioscience, 1:100) and PE-Cy7 anti-mouse CD80 antibody (clone: 16-10A1; eBioscience, 1:100) on ice for 0.5 hours. After washing with PBS, the cells were resuspended with FACS buffer and analyzed on an LSRFortessa 4-15 flow cytometer.

### GA stability in plasma

GA or OX/GA (100 parts per million) in rat plasma was prepared and maintained at 37°C. At different time points, 20-μl aliquots were collected, digested with 0.1 M HCl, and diluted with PBS. The mixture was analyzed with a 2',3'-GA ELISA kit (Cayman).

### In vivo PK

Eight-week-old SC/CD female rats were intravenously injected with OX+GA or OX/GA at a dose of 3.0 mg/kg OX and 0.36 mg GA/kg. Plasma samples were collected at 5 and 30 min and 1, 3, 5, 8, 24, and 48 hours after injection and immediately centrifuged at 604*g* for 10 min to harvest plasma samples. Plasma (20 μl) was digested with concentrated nitric acid for 24 hours and analyzed for Pt concentration by ICP-MS. For ELISA detection, 20 μl of plasma was digested in 80 μl of 0.1 M HCl solution for 10 min followed by an ELISA test using a 2,3-GA ELISA kit (Cayman).

### Biodistribution in WT, *Tmem173^−/−^*, and *Tek*^ΔSTING^ mice

Mice were subcutaneously injected with 2 × 10^6^ MC38 cells into the right flanks. When the tumors reached ~100 mm^3^, mice were intravenously injected with OX, OX-NCP, or OX/GA at a dose of 3.0 mg OX/kg and/or 0.36 mg GA/kg. Plasma, heart, brain, liver, lung, spleen, kidney, and tumor samples were collected at 3, 8, 24, 48, and 72 hours after injection after transcardiac perfusion with 20 ml of PBS. Organ weights were measured, and Pt concentrations were quantified by ICP-MS.

### Biodistribution of fluorescently labeled OX/GA

C57BL/6 mice were subcutaneously injected with 2 × 10^6^ MC38 cells in the right flanks. When the tumors reached ~100 mm^3^, the mice were intravenously injected with 100 μg of OX-pyro or OX/GA-pyro. Twenty-four hours later, tumors were harvested. Frozen tissue sections were prepared using a cryostat. The sections were fixed in acetone for 10 min at −20°C and stained with 4′,6-diamidino-2-phenylindole (DAPI) for another 10 min. The sections were then washed twice with PBS and imaged by CLSM.

For flow cytometry, the freshly harvested tumors were treated with collagenase I (1 mg/ml; Gibco, United States) for 1 hour at 37°C and ground with the rubber end of a syringe. The cells were filtered through nylon mesh filters and washed with PBS. The single-cell suspensions were analyzed by flow cytometry.

### Mechanical analysis

Uniaxial compression and stress relaxation tests were conducted for mechanical analysis of tumors on a strain-controlled dynamic mechanical analyzer (RSA-G2, TA Instruments). Tumors of 100 to 150 mm^3^ in sizes were loaded between 1.5-cm-diameter stainless steel parallel plates with an excess of buffer to maintain adequate hydration during the experiment. All tests were performed at room temperature. For uniaxial tests to quantify Young’s moduli, tumor samples were preloaded with a compression force of 0.05 N and subjected to a compressive strain with a 50% min^−1^ constant compression rate. The average Young’s moduli for the control and treated tumors were obtained from analysis of the stress-strain curves in the linear viscoelastic regions, below 5% compression. For stress relaxation to determine tumor viscoelasticity, tumor samples were preloaded with a compression force of 0.05 N and subjected to a uniaxial compressive step strain (2%). The generated compressive stress was monitored for 400 s.

### CD31 staining

C57BL/6 mice were subcutaneously injected with 2 × 10^6^ MC38 cells in the right flanks. When the tumors reached ~100 mm^3^, mice were intravenously injected with PBS, OX+GA, OX-NCP, GA-NCP, or OX/GA at a dose of 3.0 mg/kg OX and/or 0.36 mg GA/kg. Twenty-four hours later, tumors were harvested.

For immunofluorescence imaging, frozen tissue sections were prepared using a cryostat. The sections were fixed in acetone for 10 min at −20°C and stained with APC anti-mouse CD31 antibody (clone: 10F.9G2; BioLegend, 1:100), followed by DAPI for another 10 min. The slices were scanned by a Leica SP8 CLSM.

For flow cytometry, the freshly harvested tumors were treated with collagenase I (1 mg/ml; Gibco, United States) at 37°C for 1 hour and ground with the rubber end of a syringe. The cells were filtered through nylon mesh filters and washed with PBS. The cells were transferred to Eppendorf Tubes and incubated with anti-CD16/32 (clone: 93; eBioscience, 1:100) to reduce nonspecific binding to FcRs. Cells were further stained with the Alexa Fluor 647 anti-mouse CD31 antibody (clone: MEC13.3; BioLegend, 1:100). After washing with PBS, the cells were resuspended with FACS buffer and analyzed on an LSRFortessa 4-15 flow cytometer.

### Histology and IHC

Tissues were fixed with 4% paraformaldehyde for 2 to 3 days and sent to the University of Chicago core facility for embedding and processing. The slides were stained using a Leica Bond RX automated stainer. Epitope retrieval solution I (Leica Biosystems, AR9961) was used for heat treatment for 20 min. Primary rabbit monoclonal anti-mouse CD31 (Abcam, ab28364) was used for 1 hour, and the antigen-antibody binding was detected using Bond polymer refine detection (Leica Biosystems, DS9800). Slides were scanned using CRi Pannoramic SCAN 40x Whole Slide Scanner and analyzed by QuPath.

### In vitro cytotoxicity

HUVECs and/or THP-1 were seeded in 96-well plates at a density of 5 × 10^3^ cells per well for 24 hours. Cells were then treated with different concentrations of GA or LPS for another 48 hours. MC38, Panc02, BMDCs, or BMDM cells were seeded in 96-well plates at a density of 2 × 10^3^ cells per well and allowed to adhere for 24 hours. Cells were then treated with different concentrations of OX/GA for another 48 hours. Cell viability was detected by MTS assay (Promega).

### In vivo anticancer efficacy

A total of 2 × 10^6^ cells CT26 or MC38 cells were subcutaneously injected into the right flank regions of 6-week-old BALB/c (for CT26) or C57BL/6 (for MC38) WT mice, *Tmem173^−/−^* mice, or *Tek*^ΔSTING^ mice. Seven days after tumor inoculation, the mice were intravenously dosed with PBS, OX+GA, OX-NCP, GA-NCP, and OX/GA at a dose of 3.0 mg OX/kg and 0.36 mg GA/kg Q3D for up to five doses. Tumor growth was monitored by measurement with a digital caliper, where tumor volumes were calculated as follows: (width^2^ × length)/2. TGI was calculated based on the following formula

$$\text{TGI}=1-\frac{T_{\text{endpoint}}}{C_{\text{endpoint}}}\times 100\%$$

where *T*_endpoint_ and *C*_endpoint_ refer to the tumor size of treated mice and PBS control, respectively.

### Tumor challenge studies

Sixty days after the first MC38 inoculation, tumor-free C57BL/6 mice from the OX/GA group were inoculated again with 2 × 10^6^ MC38 cells subcutaneously. Naive mice were inoculated simultaneously and monitored until reaching the protocol limit. All mice were euthanized on day 60 after rechallenge.

### Spontaneous TRAMP model

B6 TRAMP mice [C57BL/6-Tg(TRAMP)8247Ng/J] breeding pairs were purchased from the Jackson Laboratory and housed and bred as instructed. The male TRAMP offspring was verified with polymerase chain reaction by Transnetyx genotyping. Male TRAMP mice (24 weeks old) were treated with PBS, OX+GA, OX-NCP, GA-NCP, and OX/GA at a dose of 3.0 mg OX/kg and 0.36 mg GA/kg Q3D for 10 doses. Thirty days after the first injection, the prostates and seminal vehicles (genitourinary tracts) were harvested and assessed against the mouse body weight. TGI was calculated based on the following formula

$$\text{TGI}=1-\frac{T_{\text{endpoint}}-N_{\text{endpoint}}}{C_{\text{endpoint}}-N_{\text{endpoint}}}\times 100\%$$

where *T*_endpoint_, *C*_endpoint_, and *N*_endpoint_ refer to the ratio between genitourinary tracts and mouse body weight of treated mice, PBS control, and WT naive mice at the treatment endpoint, respectively.

### Ultrasound imaging of orthotopic Panc02 model

General anesthesia in mice was induced using 2.5% (v/v) isoflurane/O_2_. The abdominal cavity was opened by a 1.5-cm-wide transverse laparotomy pointing slightly to the right. The head of the pancreas was identified and lifted by a cotton wool tip. A total of 1 × 10^6^ tumor cells were slowly injected into the head of the pancreas using a precooled 27-gauge needle and a precooled calibrated special syringe (Hamilton Syringe, Reno, NV, United States). To prevent leakage, a cotton wool tip was pressed onto the injection site for 30 s. The pancreas was then placed back into the abdominal cavity. The abdominal cavity was closed by a running single-layer 5-0 VICRYL suture (Ethicon, United States). Three days after inoculation, the tumors were scanned by ultrasound imaging (Prospect 3.0 Pre-Clinical Ultrasound System, S-Sharp Corporation) to determine their sizes. For survival studies, mice were followed until death or euthanized by a blinded observer when signs of morbidity were evident.

### Cellular distribution of OX/GA

MC38 tumor-bearing C57BL/6 mice (~100 mm^3^ tumor volume) were intravenously injected with 100 μg of OX-pyro or OX/GA-pyro. Twenty-four hours later, the tumors were digested with 500 μl of RPMI 1640 + 10% FBS + collagenase I (1 mg/ml; Gibco) + collagenase IV (250 μg/ml; Gibco) + deoxyribonuclease (DNase) I (50 μg/ml; Sigma-Aldrich) cocktail at 37°C for 45 min. The digests were neutralized with 4.5 ml of complete RPMI 1640 medium and gently ground and filtered through sterile cell strainers (40 μm, Fisherbrand) to obtain single-cell suspensions. The cells pellets were washed with FACS buffer, blocked by anti-CD16/32 antibody (clone: 93, 1:100) at 4°C for 25 min, and stained with fluorochrome-conjugated rat anti-mouse antibodies 1:200 (1:500 for CD45-BV421, clone: 30-F11, BioLegend) at 4°C for 45 min. Different cell types were sorted and counted based on the following markers: tumor cells (CD45^−^), myeloid cells (CD45^+^CD11b^+^), and T cells (CD45^+^CD3e^+^). The collected cells were washed and digested with nitric acid, and the intracellular Pt level was analyzed by ICP-MS. The MFI of pyro in the different types of cells was analyzed in tumor cells (CD45^−^), ECs (CD31^+^), macrophages (CD45^+^CD11b^+^F4/80^+^), DCs (CD45^+^CD11b^+^CD11c^+^), MDSCs (myeloid-derived suppressor cells, CD45^+^CD11b^+^Gr-1^+^), and T cells (CD45^+^CD3e^+^).

### CRT exposure

MC38 cells (2 × 10^5^ cells per well) were seeded in six-well plates and then cultured with PBS, OX-NCP, GA-NCP, or OX/GA at an equivalent concentration of 15 μM OX and/or 1 μM GA for 24 hours. The cells were harvested, incubated with Alexa Fluor 488–CRT antibody (1G6A7, Novus, diluted 1:100) for 0.5 hours, and analyzed by flow cytometry to identify CRT exposure.

### HMGB1 release

MC38 cells (2 × 10^5^ cells per well) were cultured in six-well plates overnight and treated with OX-NCP, GA-NCP, or OX/GA at equivalent concentrations of 15 μM OX and/or 1 μM GA for 24 hours. The media were collected and centrifuged (14,000*g*, 10 min) to obtain the supernatants. HMGB1 released from MC38 cells to the supernatants was detected by an ELISA kit (Chondrex, United States) according to the manufacturer’s instructions.

### Phagocytosis

A total of 1 × 10^5^ MC38 cells were seeded in six-well plates with a coverslip at the bottom of each well and stained with CellTrace carboxyfluorescein diacetate succinimidyl ester (CFSE) (Invitrogen, United States) for labeling. After washing excess CFSE three times with media, 2 × 10^5^ BMDCs were added to each well and then dosed with PBS, OX-NCP, GA-NCP, and OX/GA an equivalent concentration of 15 μM OX and/or 1 μM GA for 24 hours. The cells were washed with PBS and then fixed with 4% paraformaldehyde (pH = 7.2) at room temperature for 15 min. For staining BMDCs, the cells were washed with PBS and blocked by 5% FBS in PBS at room temperature for 1 hour. The cells were then incubated with 1:100 PE-eFluor610–conjugated CD11c antibody (clone: N418, eBioscience) in 1% BSA in PBS at 4°C overnight. The cells were washed with PBS and then stained with 1:3000 Hoechst 33342 in PBS for 10 min. After washing with Dulbecco’s PBS (DPBS), the coverslips were mounted on glass slides with ProLong glass antifade mountant, cured at room temperature overnight, sealed by nail polish, and observed on a Leica SP8 confocal microscope.

### In vivo DC maturation in TDLNs

MC38 tumor-bearing C57BL/6 mice were intravenously dosed with PBS, OX+GA, OX-NCP, GA-NCP, and OX/GA at a dose of 3.0 mg OX/kg and 0.36 mg GA/kg. On day 4 after injection, the TDLNs were harvested and digested at 37°C for 45 min by a cocktail of 600 μl of RPMI 1640 medium + 10% FBS + collagenase I (1 mg/ml; Gibco) + collagenase IV (250 μg/ml; Gibco) + DNase I (50 μg/ml; Sigma-Aldrich). The digests were mixed with 4.4 ml of RPMI 1640 medium, gently ground, and filtered through the sterile cell strainers (40 μm, Corning) to obtain the single-cell suspension. The cells were obtained by centrifugation (300*g*, 10 min) at 4°C. For live cell staining, the red blood cells were lysed by ACK buffer (Thermo Fisher Scientific; 2 ml per sample), and the remaining cells were washed with FACS buffer followed by staining with fluorochrome-conjugated antibodies for analysis. DCs in TDLNs were stained by CD45-BV421 (clone: 30-F11, BioLegend, 1:100), CD11b-FITC (clone: M1/70, eBioscience, 1:100), CD11c-PE-eFluro610 (clone: N418, eBioscience, 1:100), MHCII-PE (clone: M5/114.15.2, BioLegend, 1:100), and APC anti-CD86 (clone: GL1, eBioscience, 1:100).

### IFN-γ ELISPOT assay

A MultiScreenHTS IP plate (Millipore Sigma) was activated by 70% ethanol, washed with DPBS, coated with anti-mouse IFN-γ capture antibody (BD Biosciences) at 37°C for 8 hours, and blocked with sterile 1% BSA in DPBS at room temperature for 2 hours. The spleens were harvested from treated MC38 tumor-bearing C57BL/6 mice and then gently ground and filtered through sterile cell strainers to afford single-cell suspensions. Red blood cells were then lysed by sterile ACK buffer (Corning), and splenocytes were counted and seeded in the plate at a density of 2 × 10^5^ cells per well in RPMI 1640 full medium (six mice in each treatment group and each mouse with three replicates). MC38 tumor-associated peptide KSPWFTTL (KSP) was added to each well at a concentration of 10 μg/ml except for negative control wells. The splenocytes in positive control wells were directly stimulated with anti-mouse CD3Ɛ (145-2C11) and anti-mouse CD28 (37.51) antibodies (eBioscience, 1:1000). The splenocytes were incubated at 37°C for 48 hours, and the culture media were discarded. The plates were then washed and incubated with a biotinylated anti–IFN-γ detection antibody, streptavidin–horseradish peroxidase conjugate, and 3-amino-9-ethylcarbazole (AEC) substrate (BD Biosciences). The plate was air-dried and analyzed by a CTL ImmunoSpot S6 Analyzer.

### Proinflammatory cytokine detection

Mouse tumor tissues were collected 24 hours after treatment. Tumor tissues were weighted after collection, homogenized, and centrifuged at 12,000*g* for 10 min to collect the supernatants. Protease inhibitor cocktails (Thermo Fisher Scientific) were added. Inflammatory cytokines were measured using LEGENDplex Mouse Inflammation Panel (13-plex) kit (BioLegend).

### Gene expression analysis

OX/GA-treated tumors were collected at 4 days after treatment and mixed with TRIzol (Life Technologies) to extract the RNAs. Three replicates of each RNA sample were subjected to gene quantification with the nCounter PanCancer IO 360 Panel. To quantify gene expression levels, 5 μl of an RNA suspension (30 ng/μl) was first subjected to a hybridization step in a C1000 Touch Thermal Cycler (Bio-Rad, Hercules, CA, United States) according to the nCounter XT CodeSet Gene Expression Assays Protocol (NanoString Technologies). Briefly, samples were hybridized at 65°C for 18 hours in a 15-μl volume consisting of 3 μl of Reporter CodeSet (NanoString Technologies), 5 μl of hybridization buffer (NanoString Technologies), 5 μl of RNA sample, and 2 μl of Capture ProbeSet (NanoString Technologies). After hybridization, the samples were placed in a NanoString nCounter FLEX Analysis System (NanoString Technologies) for purification and immobilization in a Prep Station (NanoString Technologies) and data collection in a Digital Analyzer (NanoString Technologies).

Data were analyzed by ROSALIND (https://rosalind.bio/), with a HyperScale architecture developed by ROSALIND Inc. Normalization, fold changes, and *P* values were calculated using criteria provided by NanoString (https://nanostring.com). ROSALIND follows the nCounter Advanced Analysis protocol of dividing counts within a lane by the geometric mean of the normalizer probes from the same lane. Housekeeping probes to be used for normalization are selected based on the geNorm algorithm as implemented in the NormqPCR R library. The abundance of various cell populations is calculated on ROSALIND using the NanoString Cell Type Profiling Module. ROSALIND performs a filtering of Cell Type Profiling results to include results that have scores with a *P* value greater than or equal to 0.05. *P* value adjustment is performed using the Benjamini-Hochberg method of estimating false discovery rates. Enrichment was calculated relative to a set of background genes relevant to the experiment.

### Immune cell profiling

MC38 tumor-bearing C57BL/6 mice (*n* = 6) received indicated treatments, and the tumors were harvested on day 4 or 12 after the first injection for immune cell profiling by flow cytometry. The tumors and TDLNs were digested by 500 μl of RPMI 1640 + 10% FBS + collagenase I (1 mg/ml; Gibco) + collagenase IV (250 μg/ml; Gibco) + DNase I (50 μg/ml; Sigma-Aldrich) cocktail at 37°C for 45 min. The digests were neutralized with 4.5 ml of complete RPMI 1640 medium and gently ground and filtered through sterile cell strainers (40 μm, Fisherbrand) to obtain single-cell suspensions. The cell pellets were collected by centrifugation at 300*g* at 4°C for 5 min. For live staining, the cells were washed with ice-cold FACS buffer and stained first with LIVE/DEAD fixable yellow dead cell stain kit (Thermo Fisher Scientific, 1:1000). The cells were then washed with FACS buffer, blocked by anti-CD16/32 antibody (clone: 93, 1:100) at 4°C for 25 min, and stained with the fluorochrome-conjugated rat anti-mouse antibodies 1:200 (1:500 for CD45-BV421) at 4°C for 45 min. The antibodies, conjugated dyes, and clone numbers are listed as follows: CD45-BV421 (30-F11), CD11b-FITC (M1/70), NK1.1-PE/Dazzle 594 (PK130), F4/80-PerCP/Cy5.5 (BM8), Gr1-PE (RB6-8C5), CD86-APC (GL1), CD206-PE/Cy7 (C068C2), CD11c-PE-eFluor610 (N418), MHCII-PE (M5/114.15.2), CD3Ɛ-PE/eFluor610 (145-2C11), CD4-APC/H7 (GK1.5), CD8α-PerCP/eFluor710 (53-6.7), B220-APC (RA3-6B2), IFN-γ-APC (XMG1.2), granzyme B-FITC (GB11), and PD-1-PE-Cy7 (29F.1A12). CD45-BV421, CD206-PE/Cy7, and NK1.1-PE/Dazzle 594 were from BioLegend. Others were from eBioscience. The cells were lastly washed and resuspended in FACS buffer and analyzed on an LSRFortessa 4-15 flow cytometer.

### Statistical analysis

Group sizes (*n* ≥ 5) were chosen to ensure proper statistical analysis of variance (ANOVA) for efficacy studies. Student’s *t* tests were used to determine if the variance between groups is similar. Statistical analysis was performed using GraphPad Prism software version 7.0 (GraphPad Software, San Diego, CA, United States). Statistical significance was calculated using two-tailed Student’s *t* tests. The survival curves were analyzed by a Kaplan-Meier survival analysis with the log-rank (Mantel-Cox) test. Data in animal experiments are presented as means ± SD.
