# Supplementary material for: STING activation disrupts tumor vasculature to overcome the EPR limitation and increase drug deposition
Source: Sci Adv. 2024 Jul 17;10(29):eado0082. doi: 10.1126/sciadv.ado0082 (PMC466951; doi:10.1126/sciadv.ado0082)
Supplement: Supplementary file 1 — Figs. S1 to S26 Tables S1 and S2 [file sciadv.ado0082_sm.pdf]

Science Advances
AAAS

Supplementary Materials for

STING activation disrupts tumor vasculature to overcome the EPR limitation and increase drug deposition

Xiaomin Jiang *et al.*

Corresponding author: Wenbin Lin, wenbinlin@uchicago.edu

Sci. Adv. **10**, eado0082 (2024)
DOI: 10.1126/sciadv.ado0082

This PDF file includes:

Figs. S1 to S26
Tables S1 and S2

Supplementary Figures

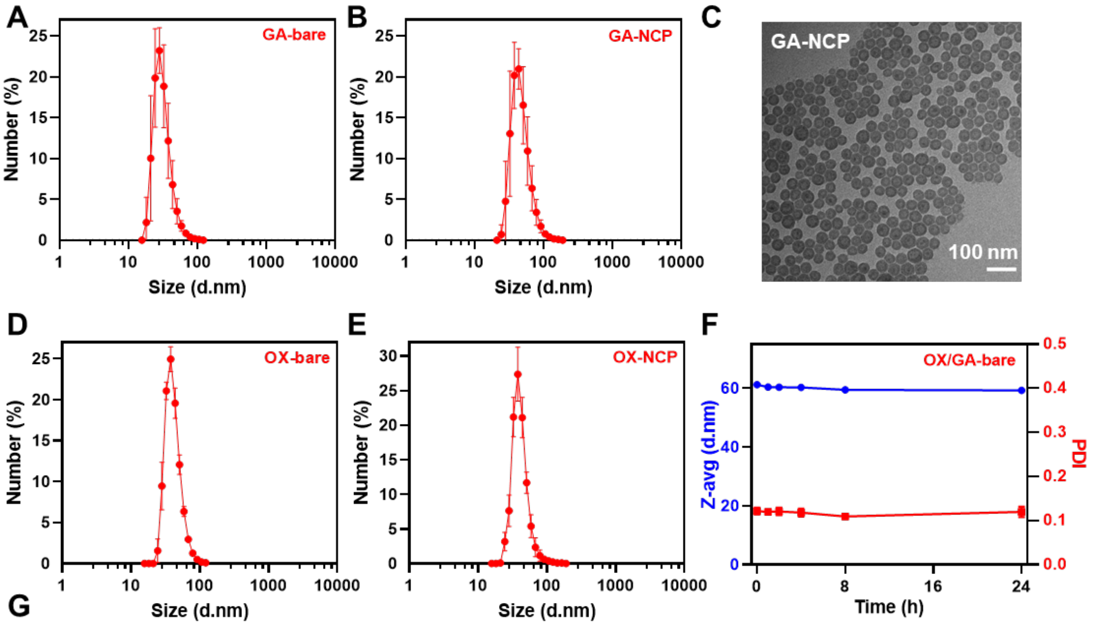

G

NCP	OX-bp	GA	Zn^{2+}	DOPA	Cholesterol	DOPC	DSPE-Peg_{2000}
OX/GA	√	√	√	√	√	√	√
GA-NCP		√	√	√	√	√	√
OX-NCP	√		√	√	√	√	√
OX/GA-bare	√	√	√	√			
GA-bare		√	√	√			
OX-bare	√		√	√			
	Core				Shell		

H

	Z-average (nm)	PDI
OX/GA	110.7 ± 1.7	0.15 ± 0.03
GA-NCP	89.4 ± 3.3	0.23 ± 0.01
OX-NCP	72.2 ± 0.5	0.25 ± 0.01
OX/GA-bare	61.2 ± 0.2	0.12 ± 0.01
GA-bare	31.7 ± 2.9	0.23 ± 0.01
OX-bare	64.5 ± 0.3	0.12 ± 0.01

Fig. S1. Characterization of control particles. (**A** and **B**) Size distributions of GA-bare (**A**) and GA-NCP (**B**) (*n* = 3). (**C**) TEM images of GA-NCP. (**D** and **E**) Size distributions of OX-bare (**D**) and OX-NCP (**E**) (*n* = 3). (**F**) Stability of OX/GA-bare in THF (*n* = 3). (**G**) Compositions of all NCPs used in this work. (**H**) Sizes and PDIs of all NCPs used in this work. Data in **A, B, D-F** are represented as mean ± SD.

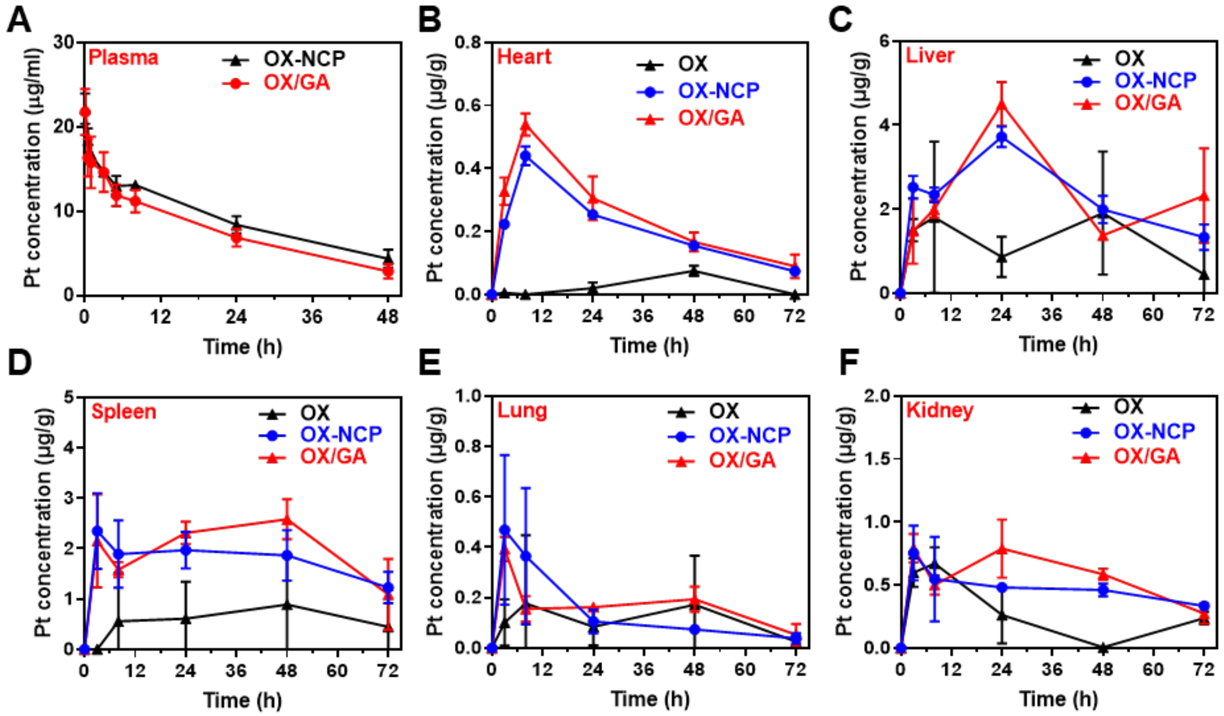

Fig. S2. Time-dependent Pt biodistribution of OX, OX-NCP and OX/GA. Organ distributions of OX, OX-NCP and OX/GA in WT mice at different time points post intravenous injection (n = 3/group). (**A**) plasma, (**B**) heart, (**C**) liver, (**D**) spleen, (**E**) lungs, and (**F**) kidneys. Data are represented as mean ± SD.

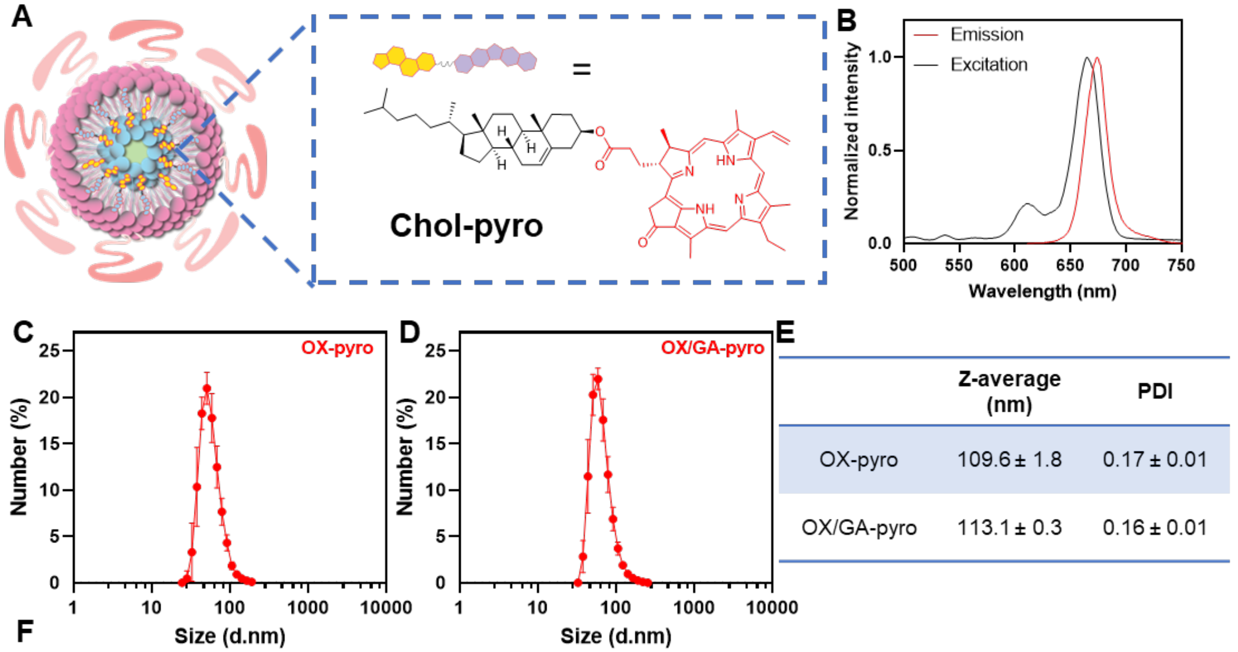

E

	Z-average (nm)	PDI
OX-pyro	109.6 ± 1.8	0.17 ± 0.01
OX/GA-pyro	113.1 ± 0.3	0.16 ± 0.01

F

NCP	OX-bp	GA	Zn^{2+}	DOPA	Cholesterol	Chol-pyro	DOPC	DSPE-Peg_{2000}
OxPt/cGAMP-pyro	√	√	√	√	√	√	√	√
OxPt-pyro	√		√	√	√	√	√	√
	Core				Shell			

Fig. S3. Characterization of fluorescently labeled NCP particles. (**A**) Scheme showing the chemical structure of Chol-pyro and its anchoring in the NCP shell. (**B**) Fluorescence spectra of Chol-pyro in THF when excited at 665 nm. (**C** and **D**) Size distributions of OX-pyro (**C**) and OX/GA-pyro (**D**) (n = 3). Data are represented as mean ± SD. (**E**) Sizes and PDIs of fluorescently labeled NCP particles. (**F**) Compositions of fluorescently labeled NCP particles.

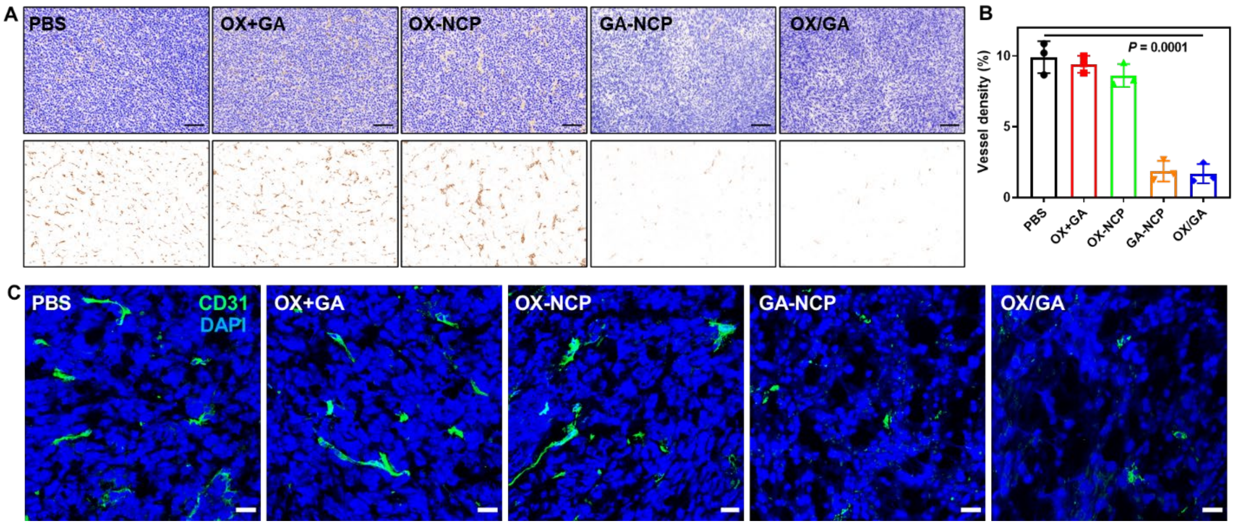

Fig. S4. IHC staining and immunofluorescence staining of tumor vessels. (**A** and **B**) CD31 IHC staining images (**A**) and quantification of vessel density (**B**) of MC38 tumors 24 hours post *i.v.* injection of OX+GA, OX-NCP, GA-NCP, or OX/GA to C57BL/6 mice at a dose of 3.0 mg OX/kg and/or 0.36 mg GA/kg (n = 3). Scale bars, 200 µm. Data are represented as mean ± SD. (**C**) CD31 immunofluorescence imaging of MC38 tumors 24 hours post *i.v.* injection of OX+GA, OX-NCP, GA-NCP, or OX/GA to C57BL/6 mice at a dose of 3.0 mg OX/kg and/or 0.36 mg GA/kg. Scale bars, 40 µm.

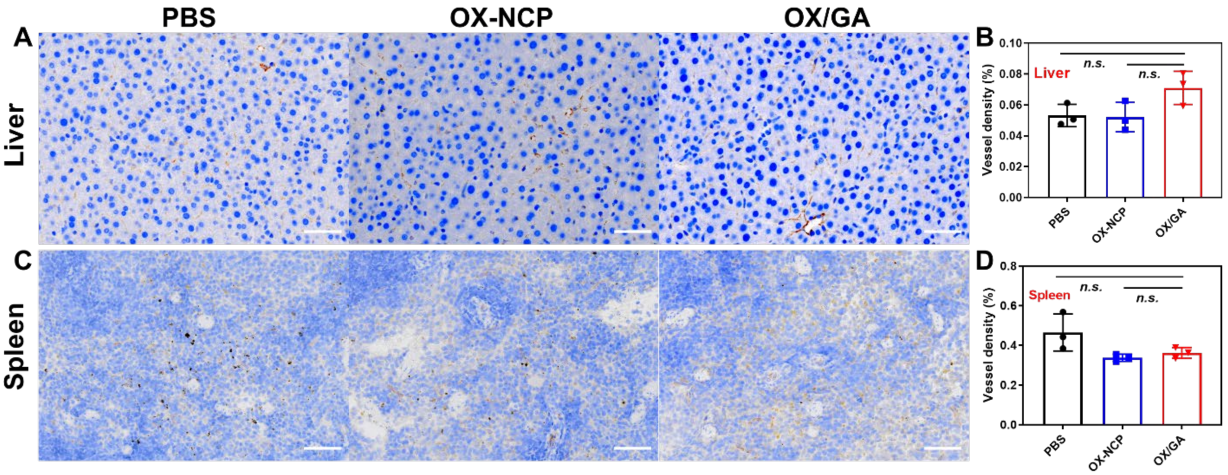

Fig. S5. IHC staining of blood vessels in the liver and spleen. CD31 IHC staining and vessel density quantification of livers (**A** and **B**) and spleens (**C** and **D**) 24 hours post i.v. injection of OX-NCP or OX/GA to C57BL/6 mice at a dose of 3.0 mg OX/kg and/or 0.36 mg GA/kg (n = 3). Scale bars, 50 µm. Data are represented as mean ± SD. The data were analyzed by one-way ANOVA with Tukey's multiple comparisons tests.

A

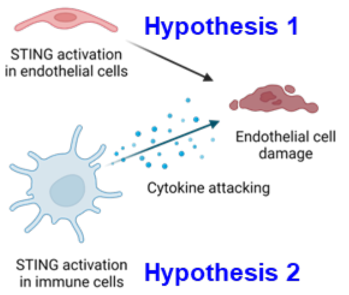

B

	ZnP	GA	GA-NCP
HUVEC	> 100	176.2 ± 23.8	22.7 ± 3.4

C

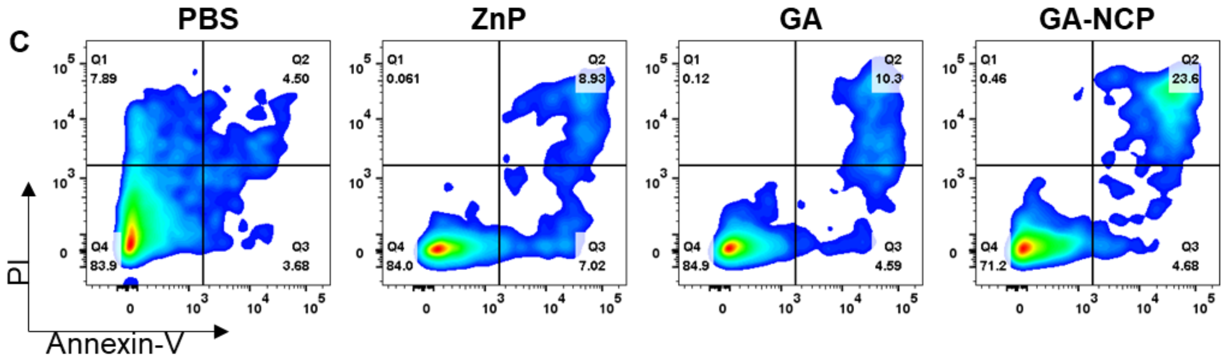

Fig. S6. Cytotoxicity of GA to endothelial cells. (**A**) Two possible mechanisms for tumor vasculature disruption by GA and GA-NCP: 1) endothelial STING activation and 2) acute elevation of cytokines secreted by STING-activated immune cell populations. (**B**) IC_{50} values (μM) of GA and GA-NCP against HUVEC. The concentration in ZnP refers to the same amount of particle as GA-NCP. (**C**) Flow cytometric analysis of HUVEC apoptosis induced by GA-NCP at a GA dose of 20 μM. The cells were stained by FITC-labelled Annexin V and propidium iodide (PI), respectively. The scheme in **A** was created with BioRender.com.

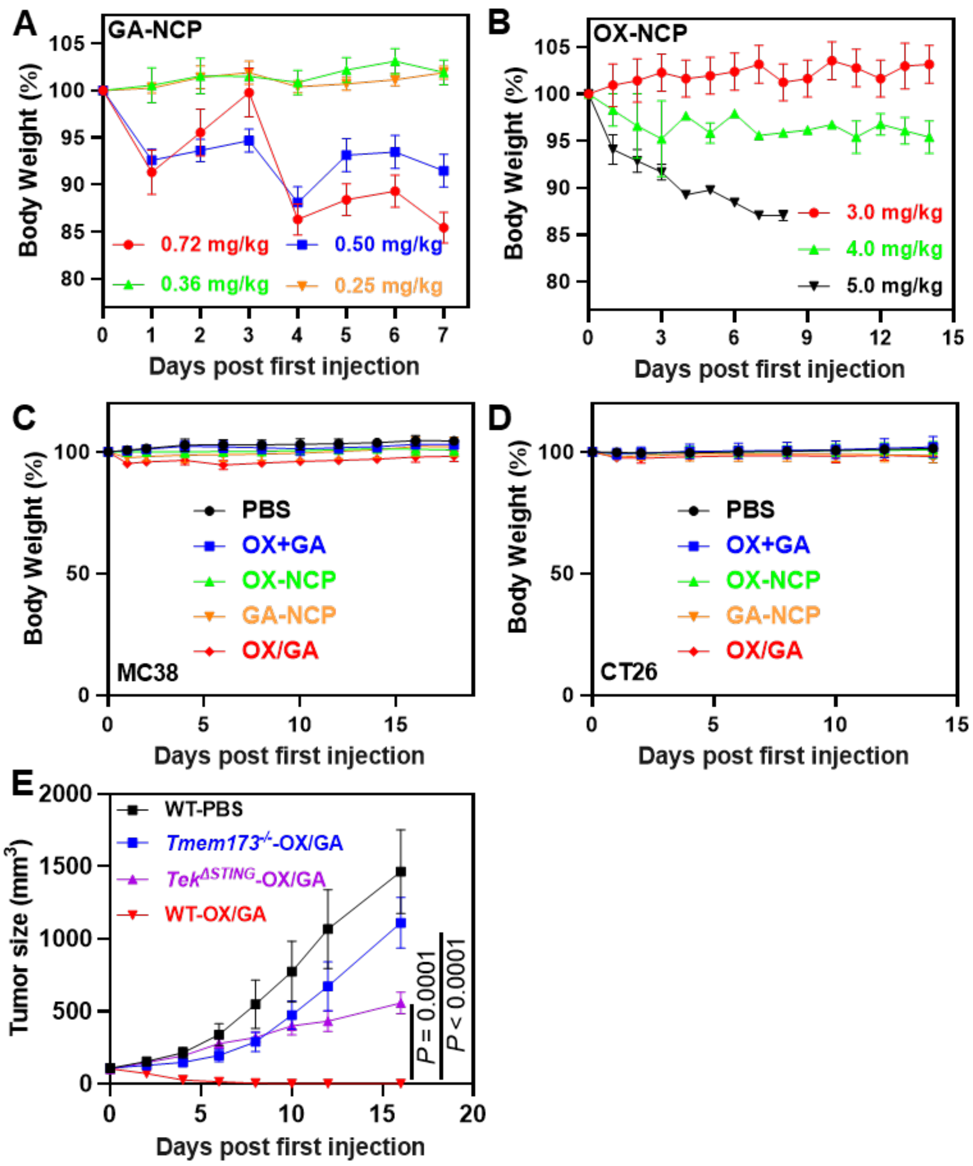

Fig. S7. (**A** and **B**) Relative body weight changes after different doses of monotherapy NCPs: GA-NCP (**A**) and OX-NCP (**B**) via intravenous injection once every three days starting on day 0. This study showed that continuous Q3D doses of 0.36 mg GA/kg or 3.0 mg OX/kg were well tolerated. (**C** and **D**) Relative body weight changes of MC38 (**C**) and CT26 (**D**) tumor-bearing mice after indicated treatments. Mice were *i.v.* injected with PBS, OX+GA, OX-NCP, GA-NCP, or OX/GA at equivalents of 3.0 mg OX/kg and/or 0.36 mg GA/kg Q3D for 5 doses ($n = 6$). Data are presented as mean ± SD. (**E**) Tumor growth curves of MC38 tumor-bearing wildtype (WT), *Tmem173*$^{-/}$ and *Tek*$^{\Delta STING}$ C57BL/6 mice after *i.v.* injected with OX/GA at 3.0 mg OX /kg and 0.36 mg GA/kg Q3D for 5 doses ($n = 6$). Data are presented as mean ± SD. The data were analyzed by one-way ANOVA with Tukey's multiple comparisons tests.

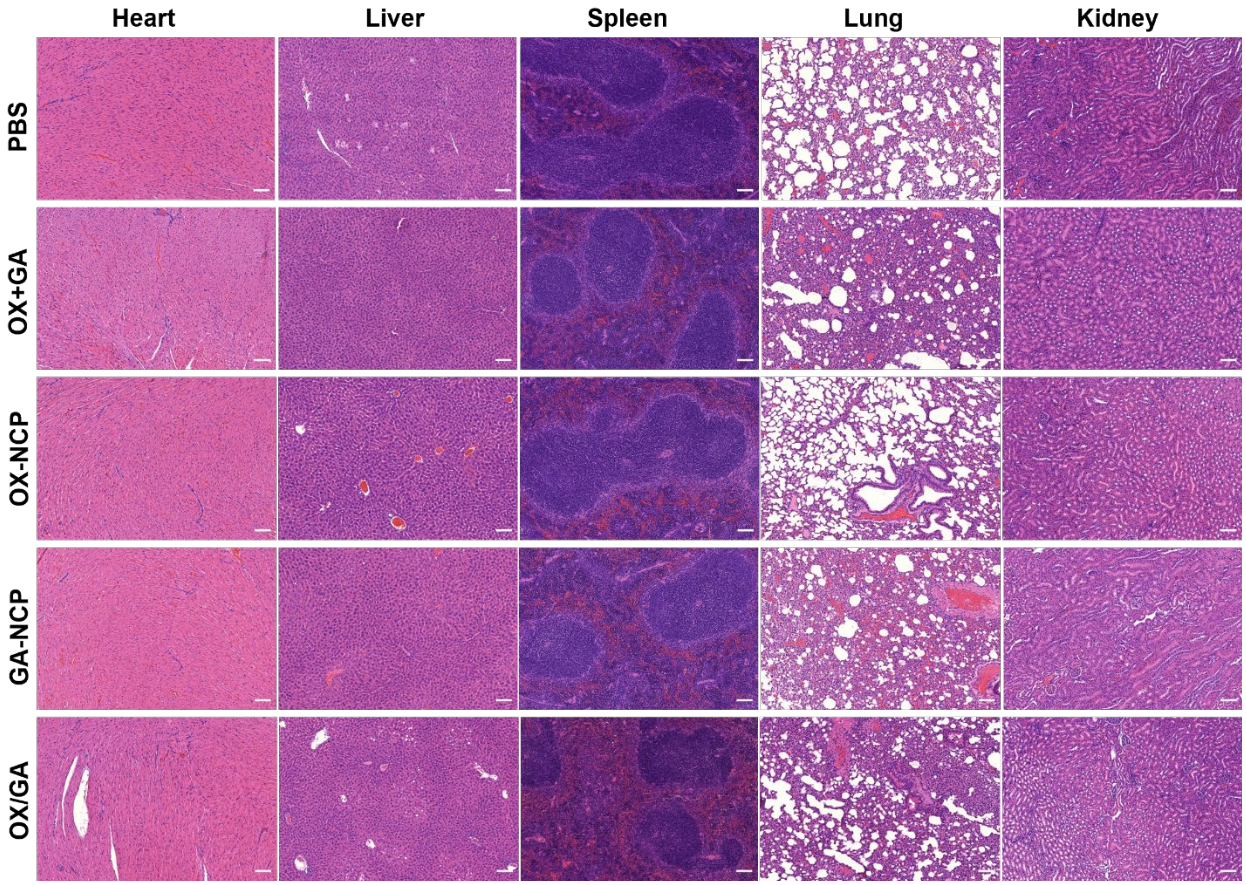

Fig. S8. H&E staining of major organs excised from MC38 tumor-bearing mice at the endpoint. Mice were *i.v.* injected with PBS, OX+GA, OX-NCP, GA-NCP, or OX/GA at equivalents of 3.0 mg OX/kg and/or 0.36 mg GA/kg Q3D for 5 doses in total ($n = 6$). Data are presented as mean ± SD. Scale bars: 100 μm.

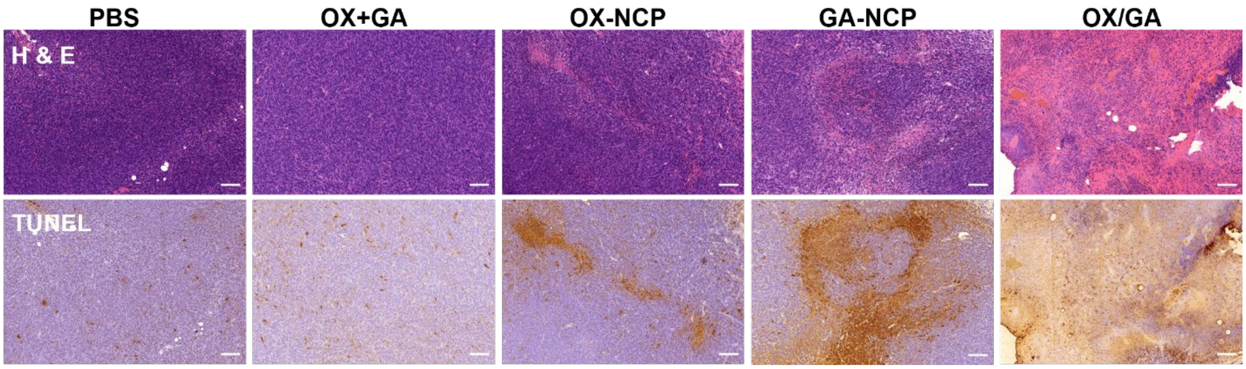

Fig. S9. H&E staining and TUNEL IHC staining of excised MC38 tumors after indicated treatments. Mice were *i.v.* injected with PBS, OX+GA, OX-NCP, GA-NCP, or OX/GA at equivalents of 3.0 mg OX/kg and/or 0.36 mg GA/kg Q3D for 5 doses in total. Scale bars: 100 μm.

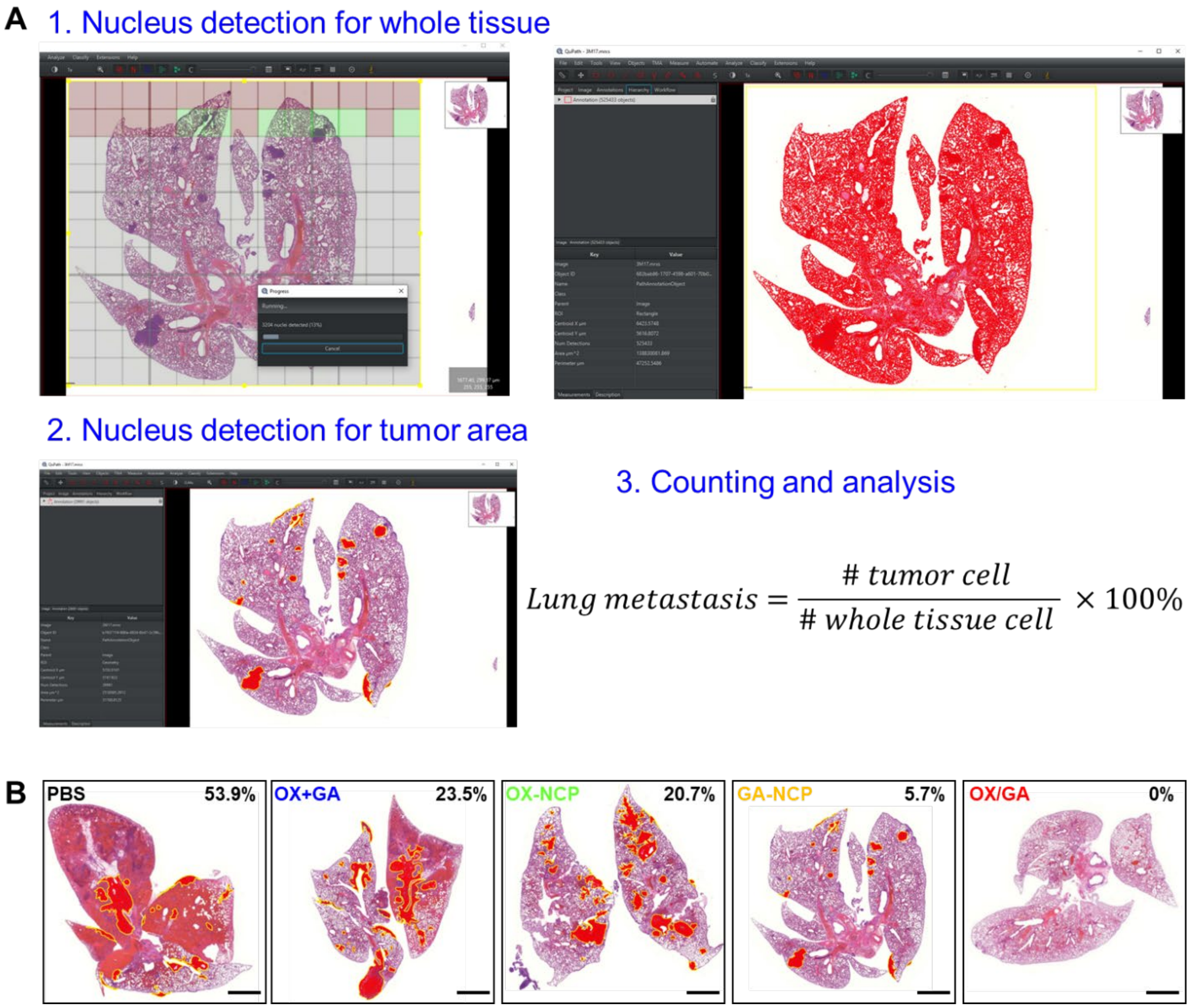

Fig. S10. Procedure (**A**) and result (**B**) of lung metastasis quantification in orthotopic 4T1 tumor-bearing BALB/c mice after *i.v.* injection of OX+GA, OX-NCP, GA-NCP, OX/GA at an equivalent dose of 3.0 mg OX/kg OX and/or 0.36 mg GA/kg Q3D for 6 doses. Scale bar: 2 mm.

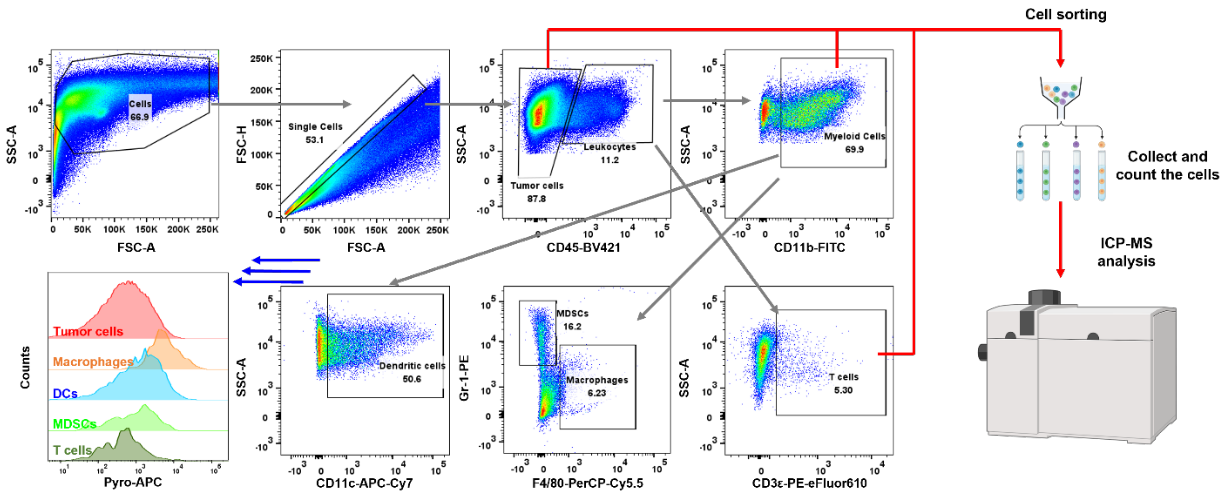

Fig. S11. Analysis process of cell distribution of OX/GA. To analyze Pt distribution, the cell suspension was stained with surface markers and then sorted using flow cytometry (red arrows). Subsequently, ICP-MS was employed to measure the Pt levels. The proportions of tumor cells, myeloid cells, and T cells were quantified using flow cytometry. In the case of fluorescently-labeled OX/GA (OX/GA-pyro) the analysis relied on assessing pyro fluorescence intensity after gating cell types based on their surface markers. The scheme was created with BioRender.com.

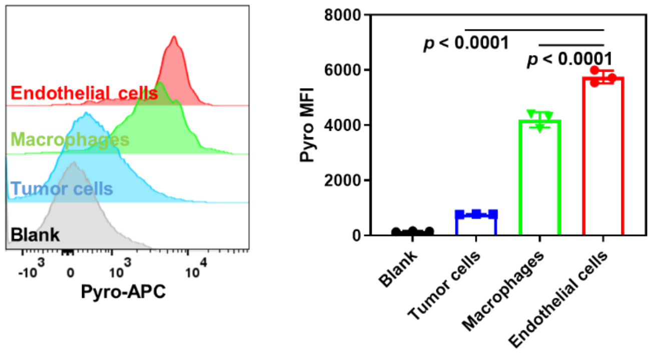

Fig. S12. Pyro MFIs in different subsets of cells in MC38 tumors 24 h post *i.v.* injection of OX/GA-pyro (n = 3). Blank: MC38 tumor cells 24 h post *i.v.* injection of PBS. The subpopulations were defined as: tumor cells, $CD45^{-}CD31^{-}$; macrophages, $CD45^{+}CD11b^{+}F4/80^{+}$; endothelial cells, $CD31^{+}$. Data are represented as mean ± SD. The data were analyzed by one-way ANOVA with Tukey's multiple comparisons tests.

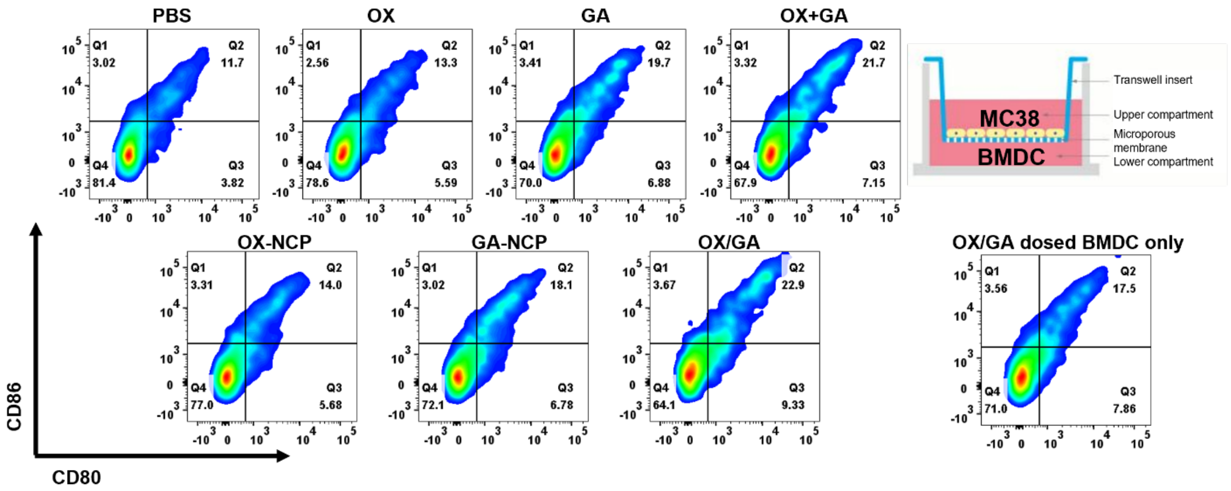

Fig. S13. In vitro DC maturation induced by OX/GA-treated MC38 cells. MC38 cells and BMDCs were seeded in the upper and lower compartments of transwells, respectively (top right). MC38 cells were incubated with different treatments at an equivalent concentration of 15 μM OX or/and 1 μM GA for 24 h and then co-cultured with BMDCs for 24 h. Bottom right: OX/GA-treated BMDCs without MC38 seeded in the transwells.

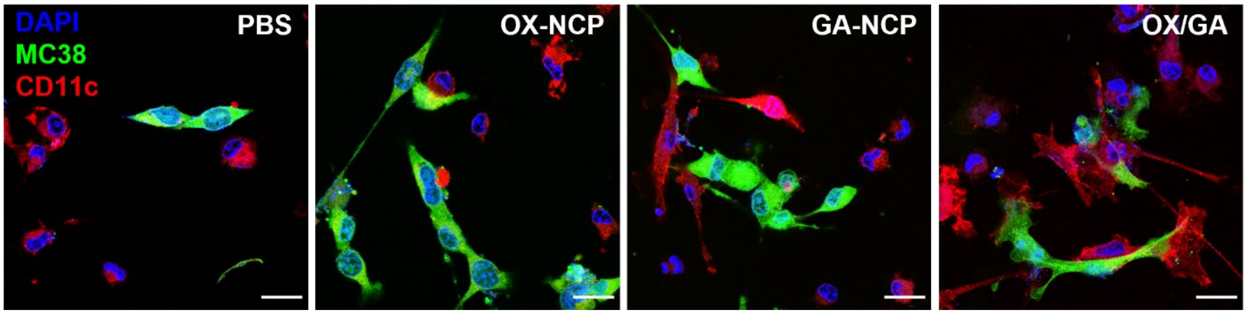

Fig S14. CLSM images showing phagocytosis of OX/GA-treated MC38 cells (green) by BMDCs (red). The MC38 cells were pre-stained with CFSE and then co-cultured with BMDCs. BMDCs were further stained with PE-eFluor610 conjugated CD11c antibody. Scale bar: 20 μm.

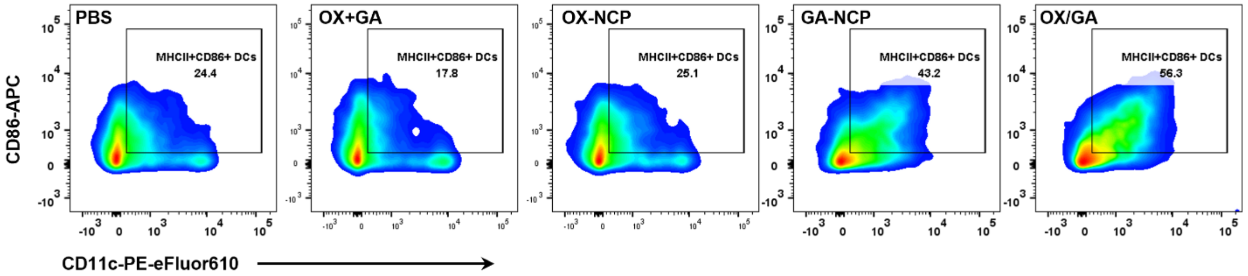

Fig. S15. Representative flow cytometry plots of mature DCs (CD11b$^+$CD11c$^+$CD86$^+$) in TDLNs from MC38 tumor-bearing C57BL/6 mice 4 days after treatments. Mice were *i.v.* injected with PBS, OX+GA, OX-NCP, GA-NCP, or OX/GA at equivalents of 3.0 mg OX/kg and/or 0.36 mg GA/kg.

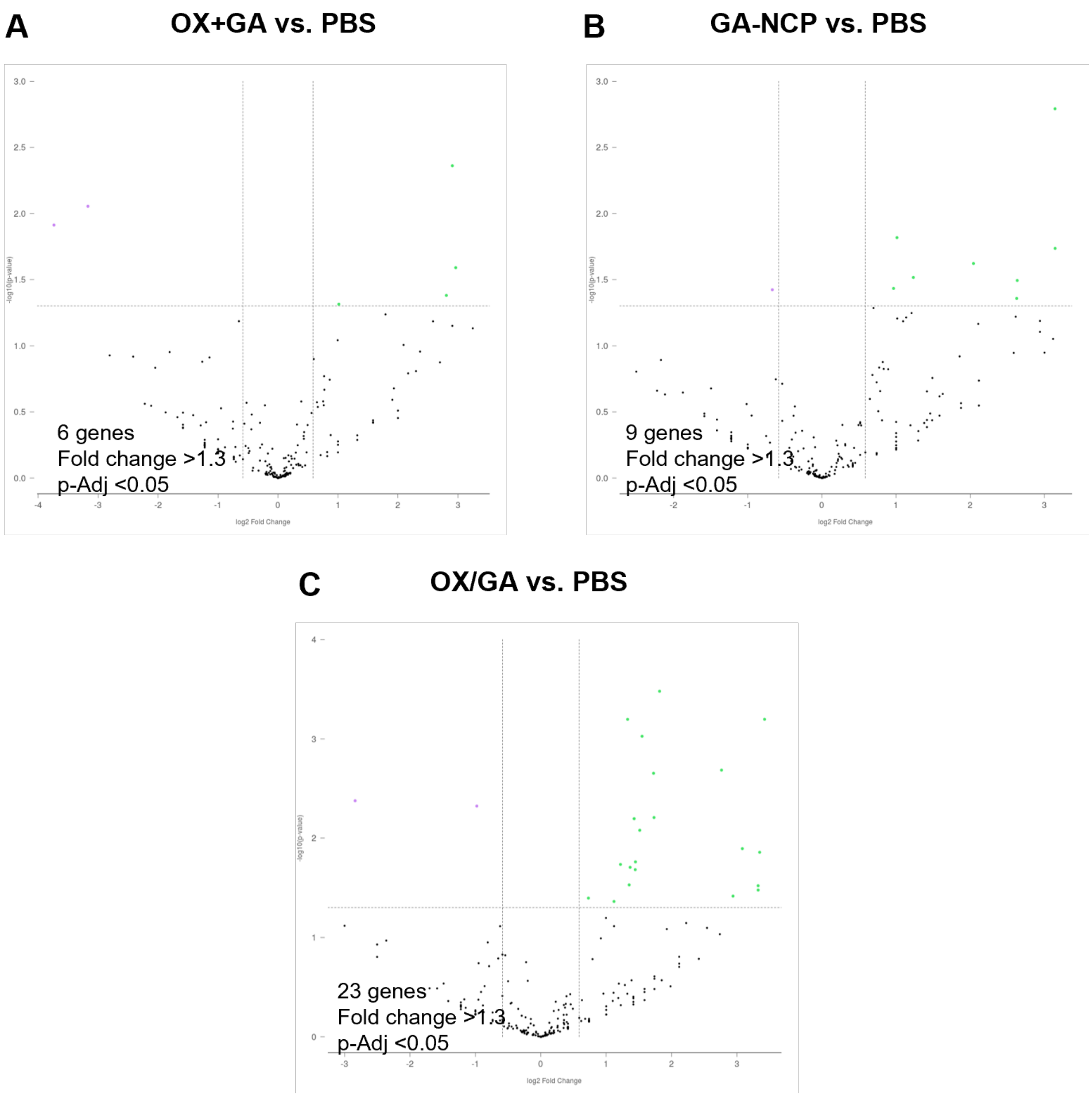

Fig. S16. Volcano plots showing the genes passing the threshold (-log(p-value)>1.3) for differential expressions of OX+GA vs. PBS (**A**), GA-NCP vs. PBS (**B**), and OX/GA vs. PBS (**C**). Upregulation is shown as green dots and downregulation is shown as purple dots.

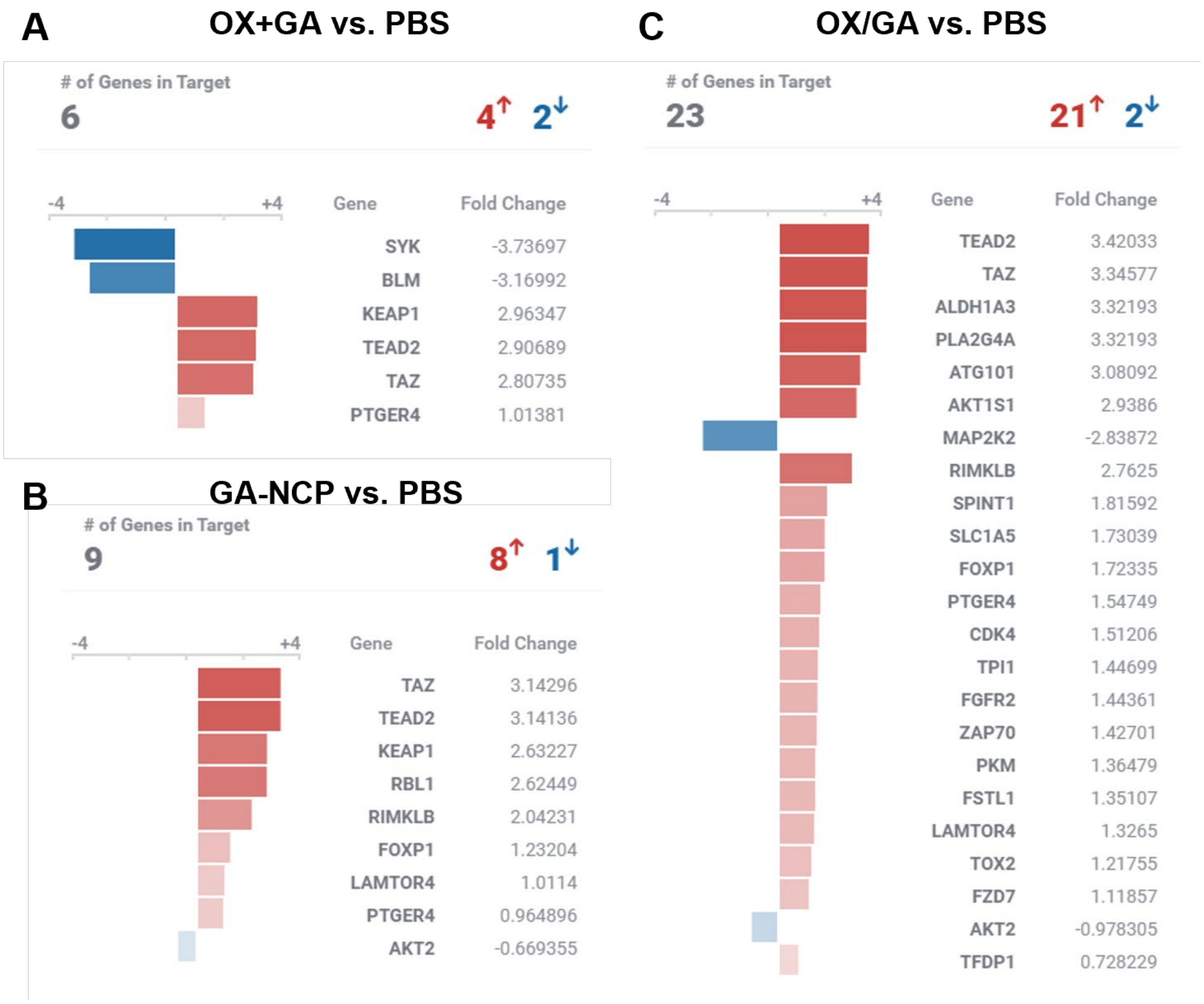

Fig. S17. Bar graphs of gene lists from NanoString. The bar graphs of the genes that have passed the threshold in **Fig. S16**, indicating differential expressions between the following conditions: OX+GA vs. PBS (**a**), GA-NCP vs. PBS (**b**), and OX/GA vs. PBS (**c**). Upregulation is denoted by red, while downregulation is represented by blue.

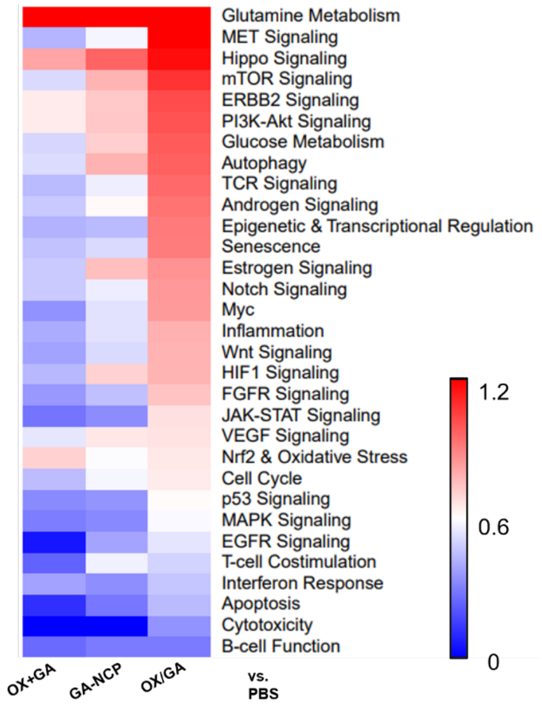

Fig. S18. NanoString gene set analysis (GSA) heatmap of directed global significance scores of OX+GA vs. PBS, GA-NCP vs. PBS, and OX/GA vs. PBS with the nCounter Tumor Signaling 360 Panel (n = 2 for OX+GA, n = 3 for the other groups). The scores quantify the degree to which a group of genes is either upregulated or downregulated in relation to the covariate. They were computed by taking the square root of the average signed squared t-statistic for the genes within the gene set. The t-statistics are obtained from the linear regression used in the analysis of differential expressions.

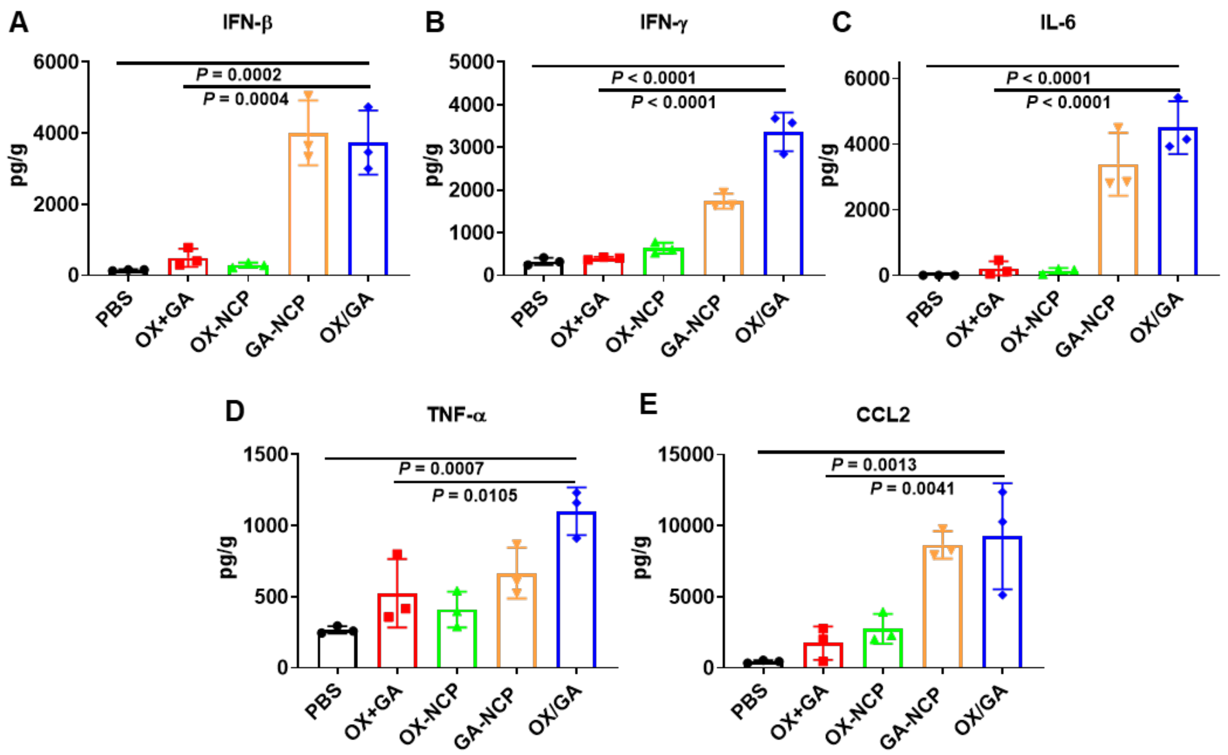

Fig. S19. Pro-inflammatory cytokines levels in MC38 tumors at 24 h after *i.v.* injection with PBS, OX+GA, OX-NCP, GA-NCP, or OX/GA at equivalents of 3.0 mg OX/kg and/or 0.36 mg GA/kg (n = 3). Data are represented as mean ± SD. The data were analyzed by one-way ANOVA with Tukey's multiple comparison test.

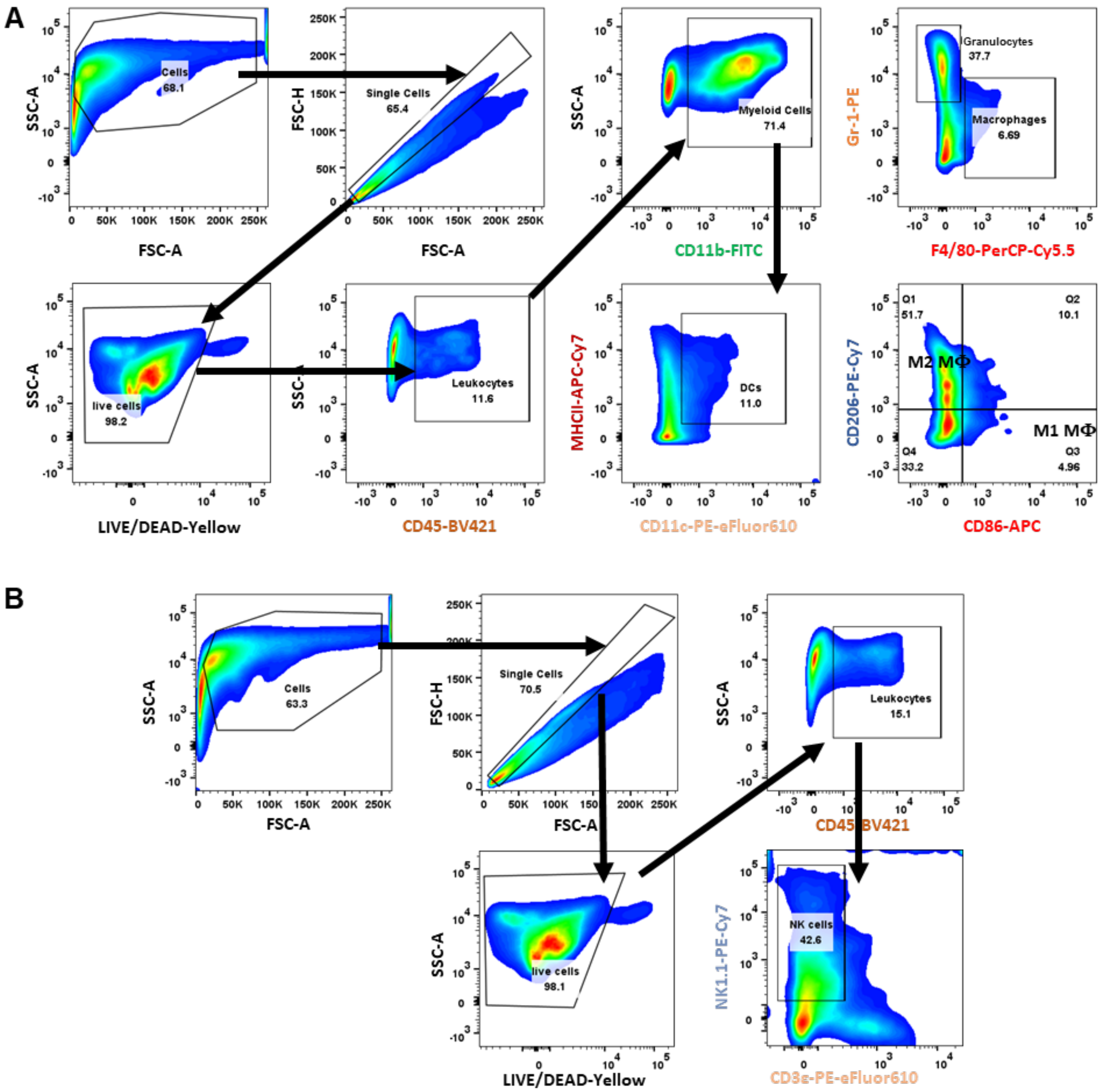

Fig. S20. (**A**) Gating strategies for leukocytes (CD45^{+}), macrophages (CD45^{+}CD11b^{+}F4/80^{+}), M1-like macrophages (CD45^{+}CD11b^{+}F4/80^{+} CD86^{+}), M2-like macrophages (CD45^{+}CD11b^{+}F4/80^{+}CD206^{+}), DCs (CD45^{+}CD11b^{+}CD11c^{+}MHC-II^{+}), and granulocytes (CD45^{+}CD11b^{+}GR-1^{+}F4/80^{-}) in **Fig. 5**. (**B**) Gating strategies for NK cells (CD45^{+}CD3ε^{-}NK1.1^{+}) in **Fig. 5**.

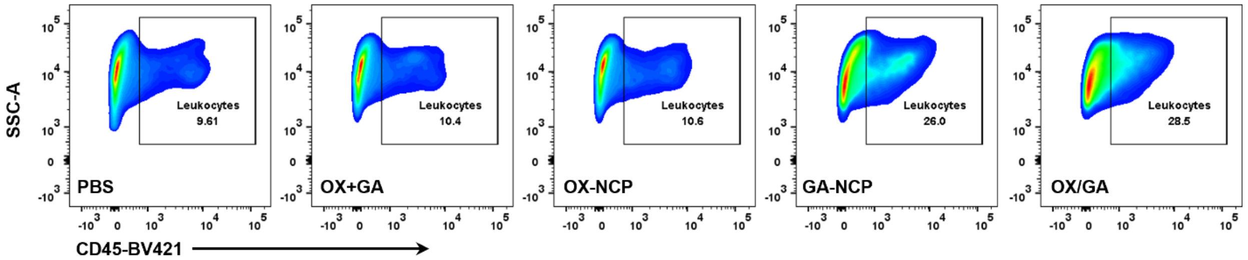

Fig. S21. Representative flow cytometry plots of leukocytes ($CD45^+$) in tumors from MC38 tumor-bearing C57BL/6 mice 4 days after treatment. Mice were *i.v.* injected with PBS, OX+GA, OX-NCP, GA-NCP, or OX/GA at equivalents of 3.0 mg OX/kg and/or 0.36 mg GA/kg.

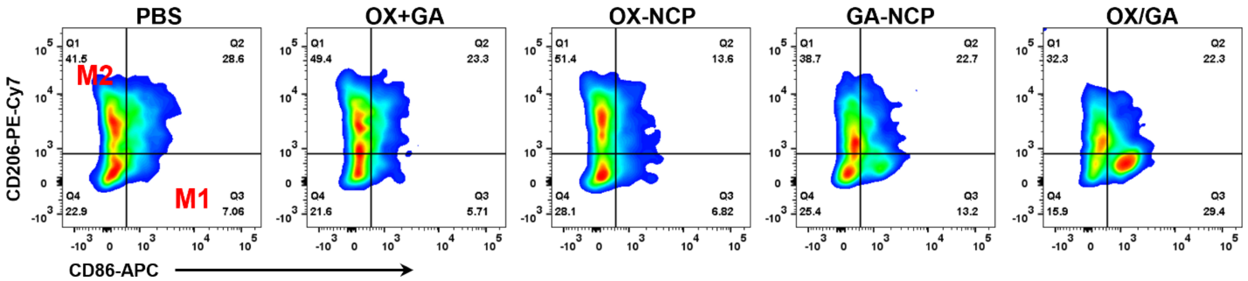

Fig. S22. Representative flow cytometry plots of M1-like macrophages ($CD45^{+}CD11b^{+}F4/80^{+}CD86^{+}$) and M2-like macrophages ($CD45^{+}CD11b^{+}F4/80^{+}CD206^{+}$) in tumor macrophages from MC38 tumor-bearing C57BL/6 mice 4 days after treatment. Mice were *i.v.* injected with PBS, OX+GA, OX-NCP, GA-NCP, or OX/GA at equivalents of 3.0 mg OX/kg and/or 0.36 mg GA/kg.

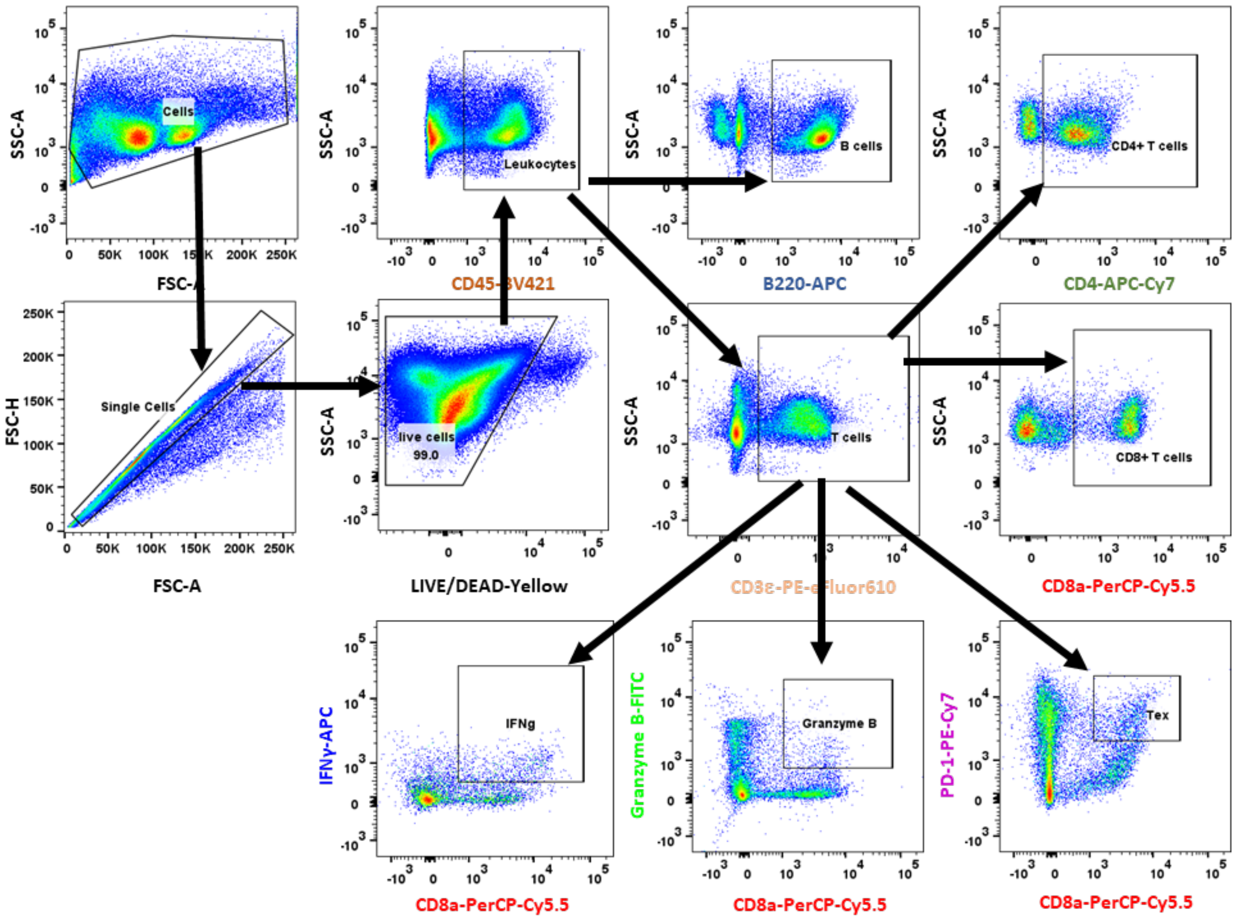

Fig. S23. Gating strategies for T cells ($CD45^{+}CD3\varepsilon^{+}$), helper T cells ($CD45^{+}CD3\varepsilon^{+}CD4^{+}$), cytotoxic T cells ($CD45^{+}CD3\varepsilon^{+}CD8^{+}$), B cells ($CD45^{+}B220^{+}$), $IFN\gamma^{+}CD8^{+}$ T cells ($CD45^{+}CD3e^{+}CD8^{+}IFN\gamma^{+}$), Granzyme $B^{+}CD8^{+}$ T cells ($CD45^{+}CD3e^{+}CD8^{+}$Granzyme B^{+}), and exhausted T cells ($CD45^{+}CD3e^{+}PD\text{-}1^{+}CD8^{+}$).

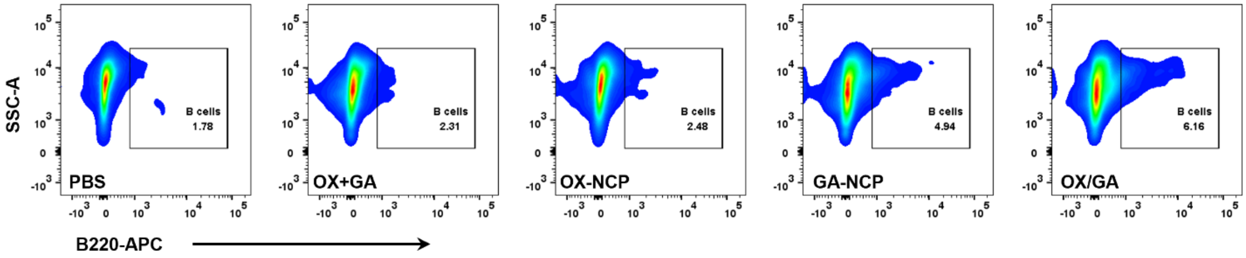

Fig. S24. Representative flow cytometry plots of B cells ($CD45^{+}B220^{+}$) in tumors from MC38 tumor-bearing C57BL/6 mice 12 days after treatment. Mice were *i.v.* injected with PBS, OX+GA, OX-NCP, GA-NCP, or OX/GA at equivalents of 3.0 mg OX/kg and/or 0.36 mg GA/kg once every three days for 4 doses.

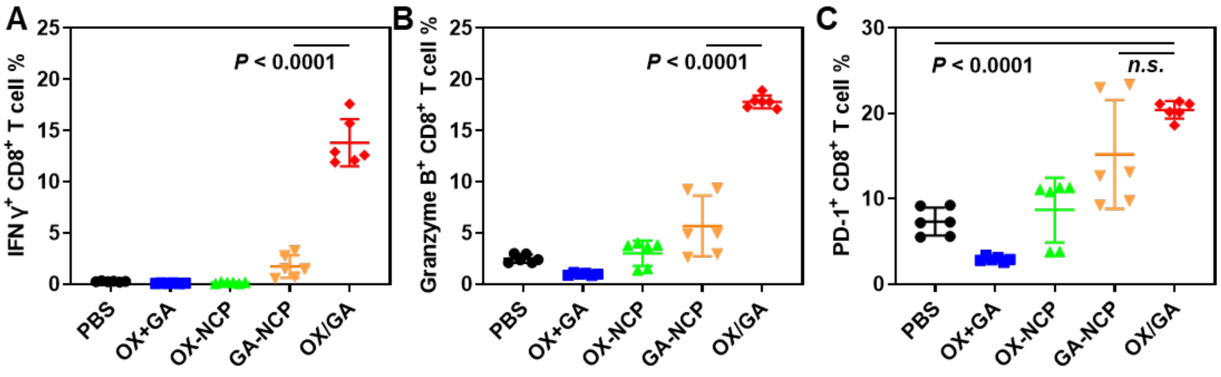

Fig. S25. Cytotoxic activity or exhaustion of $CD8^+$ T cells in tumors on day 12 post the first dose (for a total of 4 doses) as quantified by FACS. The subpopulations were defined as: (A) $IFN\gamma^+CD8^+$ T cells as $CD45^+CD3e^+CD8^+IFN\gamma^+$; (B) Granzyme B^+CD8^+ T cells as $CD45^+CD3e^+CD8^+$Granzyme B^+; (C) $PD\text{-}1^+CD8^+$ T cells as $CD45^+CD3e^+PD\text{-}1^+CD8^+$ (n = 6). Data are presented as mean ± SD. The data were analyzed by one-way ANOVA with Tukey's multiple comparisons tests.

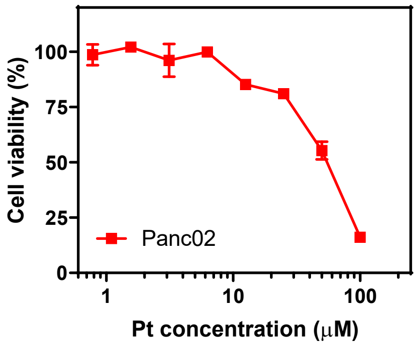

Fig S26. MTS assays of OX/GA in Panc02 cells (n = 3).

Supplementary Tables

Table S1. Plasma PK of GA in SD/CD rats following a single intravenous injection of GA or OX/GA at the dose of 3.0 mg OX/kg and/or 0.36 mg GA/kg with non-compartmental analysis.

Parameter	Unit	GA			OX/GA		
Lambda_z	1/h	2.46	±	0.44	0.04	±	0.00
t1/2	h	0.29	±	0.06	16.33	±	0.77
Tmax	h	0.08	±	0.00	0.08	±	0.00
Cmax	µg/ml	0.08	±	0.03	3.31	±	0.68
C0	µg/ml	0.09	±	0.04	3.56	±	0.85
Clast_obs/Cmax		0.00	±	0.00	0.00	±	0.00
AUC 0-t	ng/ml*h	168.22	±	3.34	42617.60	±	3966.63
AUC 0-inf_obs	ng/ml*h	171.26	±	4.55	42718.91	±	4033.82
AUC 0-t/0-inf_obs		0.98	±	0.01	1.00	±	0.00
AUMC 0-inf_obs	µg/ml*h^2	1.93	±	0.22	497.95	±	66.76
MRT 0-inf_obs	h	11.24	±	1.08	11.63	±	0.52
Vd	(mg/kg)/(µg/ml)	5.70	±	0.26	0.02	±	0.00
Cl_obs	(mg/kg)/(µg/ml)/h	0.44	±	0.01	0.00	±	0.00
Vss_obs	(mg/kg)/(µg/ml)	4.92	±	0.40	0.02	±	0.00

Table S2. Plasma PK of Pt in SD/CD rats following a single intravenous injection of OX or OX/GA at the dose of 3.0 mg OX/kg and/or 0.36 mg GA/kg with non-compartmental analysis.

Parameter	Unit	OX			OX/GA		
Lambda_z	1/h	3.36	±	0.64	0.04	±	0.00
t1/2	h	0.21	±	0.04	19.94	±	2.83
Tmax	h	0.08	±	0.00	0.08	±	0.00
Cmax	µg/ml	1.81	±	0.16	21.76	±	2.73
C0	µg/ml	2.36	±	0.34	23.05	±	2.84
Clast_obs/Cmax		0.10	±	0.03	0.13	±	0.03
AUC 0-t	µg/ml*h	2.54	±	0.32	370.43	±	42.06
AUC 0-inf_obs	µg/ml*h	3.44	±	0.19	454.76	±	71.46
AUC 0-t/0-inf_obs		0.74	±	0.12	0.82	±	0.06
AUMC 0-inf_obs	µg/ml*h^2	18.54	±	6.50	12620.77	±	3665.18
MRT 0-inf_obs	h	5.35	±	1.67	27.37	±	4.23
Vd	(mg/kg)/(µg/ml)	2.07	±	0.33	0.09	±	0.01
Cl_obs	(mg/kg)/(µg/ml)/h	0.43	±	0.02	0.00	±	0.00
Vss_obs	(mg/kg)/(µg/ml)	2.27	±	0.64	0.09	±	0.01
